# Evolution of SARS-CoV-2 caused infection in farmed minks: continuous surveillance of an 11-month outbreak at the largest Latvian mink farm

**DOI:** 10.1093/ve/veag038

**Published:** 2026-06-27

**Authors:** Ņikita Zrelovs, Edvīns Oļševskis, Jurijs Perevoščikovs, Ivars Silamiķelis, Alise Jakovele, Kristīne Lamberga, Aivars Bērziņš, Monta Brīvība, Jānis Kloviņš

**Affiliations:** Bioinformatics Core Facility & Genome Centre, Latvian Biomedical Research and Study Centre, Ratsupites street 1, k-1, Riga LV-1067, Latvia; Department of Epidemiology and Risk Assessment, Institute of Food Safety, Animal Health and Environment «BIOR», Lejupes street 3, Riga LV-1076, Latvia; Veterinary Surveillance Department, Food and Veterinary Service of the Republic of Latvia, Peldu street 30, Riga LV-1050, Latvia; Department of Infectious Disease Risk Analysis and Prevention, Centre for Disease Prevention and Control of the Republic of Latvia, Duntes street 22, k-5, Riga LV-1005, Latvia; Bioinformatics Core Facility & Genome Centre, Latvian Biomedical Research and Study Centre, Ratsupites street 1, k-1, Riga LV-1067, Latvia; Bioinformatics Core Facility & Genome Centre, Latvian Biomedical Research and Study Centre, Ratsupites street 1, k-1, Riga LV-1067, Latvia; Department of Epidemiology and Risk Assessment, Institute of Food Safety, Animal Health and Environment «BIOR», Lejupes street 3, Riga LV-1076, Latvia; Veterinary Surveillance Department, Food and Veterinary Service of the Republic of Latvia, Peldu street 30, Riga LV-1050, Latvia; Department of Epidemiology and Risk Assessment, Institute of Food Safety, Animal Health and Environment «BIOR», Lejupes street 3, Riga LV-1076, Latvia; Bioinformatics Core Facility & Genome Centre, Latvian Biomedical Research and Study Centre, Ratsupites street 1, k-1, Riga LV-1067, Latvia; Bioinformatics Core Facility & Genome Centre, Latvian Biomedical Research and Study Centre, Ratsupites street 1, k-1, Riga LV-1067, Latvia

**Keywords:** SARS-CoV-2 surveillance, spillover, one health approach, SARS-CoV-2 evolution, mink adaptation, spike protein

## Abstract

Severe acute respiratory syndrome coronavirus 2 (SARS-CoV-2), responsible for the Coronavirus Disease of 2019 (COVID-19) pandemic, can productively infect a variety of animal hosts. The evidence suggests that both wild and farmed animal populations supporting continuous transmission present us with novel concerning genotypes of SARS-CoV-2. This is especially worrisome within large and dense populations of farmed animals, such as minks, for which spillbacks of ‘mink genotypes’ to the human population have been recorded on numerous occasions. In this study, we present the results of continuous clinical, virological, serological, and genomic surveillance during the period of 11 months of the SARS-CoV-2 outbreak at the largest Latvian mink farm with ˃300 000 animals. Using the One Health approach, the COVID-19 status of minks and farm workers was constantly monitored during the surveillance period. The presence of the SARS-CoV-2 genome was confirmed in 299 minks and 32 farm workers during the outbreak. The phylogenetic analysis of 188 mink and 14 human SARS-CoV-2 isolates linked to the farm provided insight into the evolution of the virus *in situ* and contributed to epidemiological investigation. The results revealed that SARS-CoV-2 lineage B.1.177.60 was initially introduced to the mink farm by an infected farm worker between 17 February and 23 March 2021, and subsequently spread among the minks. Surveillance in the affected farm showed fluctuating virus circulation. Although the average seroprevalence in samples taken from live minks was 76.92%, a fluctuating course of infection was observed from April 2021 to March 2022. Despite the implementation of strict preventive and control measures at the farm, several additional SARS-CoV-2 strain introductions were identified during 11 months. The initial introduction of a common viral strain circulating among the people at the time soon resulted in the co-circulation of multiple sister sublineages that have evolved some concerning spike protein amino acid substitutions. Subsequent other lineage introductions into the farm were not able to spread among minks. Several independent cases of farm workers infected with genotypes restricted to this mink farm were documented throughout the study timeframe. However, the ‘Latvian mink genotypes’ were not detected in the general human population beyond epidemiologic association with the given mink farm.

## Introduction

Although severe acute respiratory syndrome coronavirus 2 (SARS-CoV-2) is not the first zoonotic coronavirus known to infect humans (Ye et al. 2020), since its emergence in late 2019 (Wu et al. 2020, Zhou et al. 2020), it quickly became the most globally troublesome coronavirus to date as the etiological agent responsible for the Coronavirus Disease of 2019 (COVID-19) pandemic and its ongoing consequences (WHO 2022). While the exact origins of the virus are not yet unambiguously elucidated, analysis of the early epidemiological evidence points to likely zoonotic event(s) linked to the wildlife markets in Wuhan, China (Holmes et al. 2021). Specifically, the most recent report by the World Health Organization’s (WHO) Scientific Advisory Group for the Origins of Novel Pathogens, published on 27 June 2025 (SAGO 2025), reaffirms the Huanan Seafood Market in Wuhan as the location associated with the human infections responsible for the wide spread of the virus to other regions in China and beyond (Worobey et al. 2022, Crits-Christoph et al. 2024, Liu et al. 2024b). The fact that bat (*Rhinolophus affinis*) coronavirus RaTG13 is ~96.1% identical to SARS-CoV-2 (Zhou et al. 2020) in regard to its genomic sequence, immediately hints that bats are quite likely to have been a natural host for its progenitor virus, as was the case for other coronaviruses identified previously (Drexler et al. 2014). Moreover, a later study (Temmam et al. 2022) identified several more closely-related coronaviruses reconstructed from *Rhinolophus* bat material sampled in Laos, including the coronavirus BANAL-52, that demonstrated the highest genome identity (96.8%) recorded among other coronaviruses to SARS-CoV-2 so far. However, in contrast to previous epidemic-causing severe acute respiratory syndrome coronavirus (SARS-CoV) and Middle East respiratory syndrome coronavirus (MERS-CoV) betacoronaviruses, where there is a consensus on an intermediate host from which the virus might have spilled into the human population [palm civets and dromedary camels, respectively (De Wit et al. 2016)], for SARS-CoV-2, the intermediate host (if any) is still a matter of considerable debate (Zhao et al. 2020, Pekar et al. 2022, Schindell et al. 2022).

At the end of June 2025, ˃17 million different SARS-CoV-2 isolate complete sequences were available through the Global Initiative on Sharing All Influenza Data (GISAID) ``EpiCov'' database (Khare et al. 2021). Although the dominant majority of the sequences are the result of SARS-CoV-2 genomic surveillance among human populations being performed in many countries around the world, a number of viral isolates associated with non-human hosts have been sequenced and publicly shared as well. For example, on 22 June 2025, SARS-CoV-2 genome sequences derived from either natural or experimental infection of mustelids, canines, felines, primates, rodents, and deer are available at the EpiCoV database, indicating a rather wide susceptible host range of SARS-CoV-2 (Bashor et al. 2021, Hobbs and Reid 2021, Prince et al. 2021, Sharun et al. 2021, Hale et al. 2022). A part of these represent sequences of SARS-CoV-2 isolates responsible for incidental natural infections of domestic animals such as cats [infections of which were also documented in Latvia (Mūrniece et al. 2024)], dogs, and even hamsters, as well as captive wildlife (e.g. isolated sporadic infections in various zoo animals) by their owners or handlers, without an ensuing sustained intra-species transmission and prolonged host-specific adaptation (Qiu et al. 2022). Of more interest, however, are the sequences associated with the farmed species of animals and wildlife capable of sustaining intra-species transmission for prolonged periods of time, best represented by the relatively amply sequenced isolates from the mink (*Neovison vison*) farming environment and free-ranging white-tailed deer (*Odocoileus virginianus*), respectively (Tan et al. 2022). Moreover, a sequence-analysis-based hypothesis suggesting that the progenitor of the Omicron variant of concern (VOC), which was formerly the dominant lineage driving the COVID-19 pandemic, might have originated in mice was once proposed (Wei et al. 2021), in addition to the arguably more commonly accepted hypothesis of its intra-host evolutionary origin in an immunocompromised subject (Liu et al. 2024a). This highlights the potential of a prolonged SARS-CoV-2 transmission in non-human animal host populations to fuel the selection of viral lineages that could and still can shape the course of the COVID-19 pandemic in case of spillback events that establish transmission in the global human population. Increased attention towards the possible non-human reservoirs of the virus in line with the ‘One Health’ approach is of paramount importance for thorough surveillance efforts that would facilitate risk assessment and timely zoonotic spillover mitigation strategies.

The majority of the publicly available SARS-CoV-2 whole-genome isolate sequences derived from non-human hosts originate from the infections of farmed minks at numerous locations throughout Europe and the USA, making up around 51% of the total non-human SARS-CoV-2 isolate genomes available at GISAID on 22 June 2025. The earliest polymerase chain reaction (PCR)-confirmed SARS-CoV-2 infections of minks are traceable back to April 2020 in two mink farms located in the province of North Brabant in the Netherlands (Oreshkova et al. 2020). It was noted that the SARS-CoV-2 infection in minks was associated with the development of respiratory symptoms, eventually leading to increased mortality among the farmed animals, with interstitial pneumonia ruled as the main cause of death (Molenaar et al. 2020, Oreshkova et al. 2020). Soon after, infections in farmed minks were also reported in Denmark (Hammer et al. 2021), Poland (Rabalski et al. 2021), USA (Eckstrand et al. 2021), and Italy (Moreno et al. 2022). The geographically closest to Latvia instances of SARS-CoV-2 infections in farmed minks were recorded in multiple Lithuanian mink farms (Smičius et al. 2026). Moreover, cases of animal-to-human transmission were recorded at several mink and human interface sites (Hammer et al. 2021, Lu et al. 2021, Munnink et al. 2021, Smičius et al. 2026).

The genomic diversity of the current publicly available mink-derived SARS-CoV-2 isolate sequences implies that little to no ‘mink-specific’ adaptations were necessary for the virus to successfully cross the species border and establish itself in minks on multiple independent occasions. Although upon the introduction of SARS-CoV-2 to a mink population, some of the specific mutations recurrently arise as a sign of convergent evolution (e.g. Spike Y453F, F486L, N501T, among others), regardless of the founding lineage, thus likely providing benefits to the adaptation of SARS-CoV-2 specifically to minks (Tan et al. 2022, Zhou et al. 2022).

The first case of a person testing positive for SARS-CoV-2 infection in Latvia was registered on 2 March 2020, 9 days before the declaration of the pandemic by the WHO. Reacting to the introduction of the virus to Latvia, a continuous sequencing campaign was launched immediately in collaboration with the local COVID-19 testing laboratories. The initial aim of the Latvian SARS-CoV-2 genomic surveillance program was to sequence and publicly share as many local viral isolate genomes as possible, which resulted in the first full-length SARS-CoV-2 isolate genome sequences from the Baltic region being made publicly available in early April 2020 (Zrelovs et al. 2021).

As the pandemic unfolded, the increasing incidence of COVID-19 cases in the country led to the modest local next-generation sequencing (NGS) capacities being overwhelmed already in mid-autumn of 2020. This prompted us to focus a significant part of the local genomic surveillance efforts on the investigation of outbreaks of particular interest. One such outbreak was a result of the introduction of the SARS-CoV-2 isolate representing the B.1.177.60 lineage common in Latvia at the time into the largest Latvian mink farm. The affected mink farm was put under restrictions and strict surveillance, which revealed the fluctuating course of infection in minks, allowed tracking of the ongoing evolution of SARS-CoV-2 mink isolates circulating at the farm, and also confirmed the infection of several farmworkers by the viral genotypes observed uniquely in minks hosted at the farm. While the efforts have shown the emergence of some concerning mutations, what could be called the ‘Latvian mink SARS-CoV-2 genotypes’ have not been detected in the general human population outside the COVID-19-affected farm located in a remote area of a sparsely populated municipality.

## Materials and methods

### Outbreak description and sampling of minks

In January 2021, the Food and Veterinary Service (FVS) developed a surveillance plan for the early detection of the SARS-CoV-2 virus genome in mink farms. The plan was based on passive surveillance and targeted weekly reporting on mink mortality and testing of at least one dead animal per week. The first samples were submitted for testing in the third week of 2021.

The first presence of SARS-CoV-2 in farmed minks in Latvia was confirmed on 10 April 2021 in a commercial farm with 64 000 breeding minks, from a sample collected several days before. The farm consists of two parts (Part A with four sectors and Part B with two sectors), and minks were kept outdoors in long, narrow open-sided sheds, allowing for light and air circulation. The sheds are around 4 m wide and run longitudinally, with roof panels to protect against the sun and rain. There are ~800 minks kept in cages in one shed (one mink per cage). The farm layout scheme is provided in Supplementary Fig. S1. There were no specific mandatory biosecurity requirements for mink farms set by the national legislation before June 2021. The affected farm was the largest in Latvia, keeping 64% of the total number of farmed minks in Latvia. Since 10 April 2021, the affected farm was placed under restrictions, official surveillance, and strict biosecurity measures, which were in place till 4 March 2022 (defined as the surveillance period). Biosecurity measures for all mink farms included a legal requirement to elaborate and implement a biosecurity plan, covering measures to reduce the risk of introduction of SARS-CoV-2 in the farm, e.g. the use of face masks, regular cleaning and disinfection of boots and clothing, as well as the regular screening of health status of the farm workers, among others.

To follow the epidemiological situation in this mink farm, FVS collected mink mortality data (weekly) and carried out sampling and weekly testing of dead minks for the presence of SARS-CoV-2. Whole carcasses of dead minks were sent to the Institute of Food Safety, Animal Health and Environment (BIOR), where oropharyngeal swabs were taken. Overall, 689 dead minks were tested for the presence of the SARS-CoV-2 genome.

Monthly clinical surveillance visits at the affected farm performed during the whole surveillance period did not reveal increased mortality or any clinical signs in minks. Blood samples from live minks were taken during these visits to follow the antibody response and circulation of SARS-CoV-2. The number of animals to be sampled was calculated to detect at least 10%–20% seroprevalence with 95% confidence interval. Live minks to be sampled were selected randomly to represent all spatial sectors within the farm. In total, 238 oropharyngeal swabs and 238 blood samples were taken from live minks for the detection of the SARS-CoV-2 genome and antibodies, respectively. Oropharyngeal swabs were collected using the UTM® Universal Transport Medium™ (Copan Diagnostics), with transport medium from the original kit applied according to the manufacturer’s instructions. Proportions of positive samples from the total number of samples were calculated and presented as virus prevalence and seroprevalence, respectively. The numbers of samples taken from dead and alive minks during the surveillance period are presented in Table 1.

**Table 1:** The number of dead and live minks tested for the presence of the SARS-CoV-2 genome (RT-PCR) and the presence of antibodies (ELISA), as well as positive results.

**SARS-CoV-2 testing results in minks**													
**Timeline (year/month)**	**2021**									**2022**			**Total**
	**April**	**May**	**June**	**July**	**August**	**September**	**October**	**November**	**December**	**January**	**February**	**March**	
**Tested dead minks (*n*)**	33	10	12	31	38	50	59	223	64	74	76	19	689
Dead RT-PCR-positive (*n*)	27	9	1	20	10	6	41	147	1	5	2	0	269
Virus prevalence (%) in dead minks	81.82	90.00	8.33	64.52	26.32	12.00	69.49	65.92	1.56	6.76	2.63	0.00	
**Tested live minks (*n*)**	11	25	20	0	20	20	55	0	29	29	29	0	238
Live seropositive (*n*)	8	17	15		20	15	35		23	25	21		179
Live RT-PCR-positive (*n*)	8	0	0		0	9	13		0	0	0		30
Seroprevalence (%) in live minks	72.73	68.00	75.00		100.00	75.00	63.64		79.31	86.21	72.41		
Virus prevalence (%) in live minks	72.73	0.00	0.00		0.00	45.00	23.64		0.00	0.00	0.00		

Virus prevalences and seroprevalences are indicated in blue and green, respectively.

### Screening of mink farm workers

During the 2021–2022 period, Latvia implemented a population-wide testing approach based on regulatory legislation. This system included testing of patients with respiratory infection symptoms both in outpatient settings (upon referral from general practitioners) and in hospitals. A mandatory component of the system was the isolation and testing of identified contacts. In most cases, SARS-CoV-2 RNA detection was used as the primary testing method. In addition to these measures, regular screening of healthy individuals belonging to risk groups was carried out. Until the end of 2021, weekly screening of mink farm workers was performed using a saliva-based method for detecting SARS-CoV-2 RNA.

### Diagnostic tests

#### Detection of the SARS-CoV-2 genome

After collection, primary sample processing was performed, and samples were stored at +4°C until initial testing. RNA was extracted either with the IndiSpin QIAcube HT Pathogen Kit on the QIAcube HT instrument (Indical Bioscience) or manually using the IndiSpin Pathogen Kit (Indical Bioscience), following the manufacturer’s protocols. Real-time reverse transcription polymerase chain reaction (RT-PCR) assays were performed in accordance with the protocol for diagnostic detection of 2019-nCoV by real-time RT-PCR described by Corman et al. 2020 (Charité–Universitätsmedizin Berlin, 17 January 2020).

Specimens and extracted RNA were aliquoted and stored at −20°C until shipment to the Latvian Biomedical Research and Study Centre for further processing.

#### Detection of SARS-CoV-2-specific antibodies

Mink blood serum samples were tested for the presence of specific SARS-CoV-2 antibodies. A commercial enzyme-linked immunosorbent assay (ELISA) kit ID Screen SARS-CoV-2 Double Antigen Multi-species (ID.Vet, France) was used. The wells of this ELISA assay are coated with purified N protein recombinant antigen, which enables the detection of specific antibodies against the nucleocapsid of the SARS-CoV-2 virus. The sample testing procedure was carried out according to the manufacturer’s instructions. Briefly, 25 μl of dilution buffer and 25 μl of the investigated mink serum sample were added to the wells and incubated for 45 min at 37 (±2)°C. Then the wells were washed five times with 300 μl of the wash solution in each well at each washing time. After washing, 100 μl of diluted conjugate was added, and the plate was incubated for 30 min at 21 (±5)°C. The washing step was repeated, and 100 μl of substrate solution was added to the wells and incubated for 20 min at 21 (±5)°C. The reaction was stopped by adding 100 μl of stop solution. The Multiskan FC Microplate Photometer (Thermo Scientific, Finland) was used at a wavelength of 450 nm for optical density measurements. Subsequently, the acquired measurements for each sample were expressed as a fraction in percentage from the positive control (S/P%). Samples with an S/P% number ≥60% were considered positive for the presence of SARS-CoV-2-specific antibodies.

#### SARS-CoV-2 isolate sequencing

SARS-CoV-2 isolate sequencing was carried out in line with the previously reported sequencing framework established during the first coronavirus wave in Latvia (Zrelovs et al. 2021). Both Illumina and MGI platforms were used, depending on the ready availability of reagents.

#### Raw sequencing data processing

For MGI read datasets, we used the MGI-developed SARS-CoV-2_Multi-PCR_v1.0 pipeline available publicly [R&D, MGI Tech bioinformatics (2020) 2024]. Briefly, Adapter clipping and read trimming were performed using SOAPnuke [v1.5.6 (Chen et al. 2018b)], clean reads were aligned against the SARS-CoV-2 isolate Wuhan-Hu-1 reference genome MN908947.3 a.k.a. NC_045512.2 with bwa mem [v0.7.17-r1188 (Li 2013)], and primer sequences were clipped from aligned reads using the Cut_Multi_Primer.py script from the pipeline. Variant calling was performed with freebayes [v1.3.4 (Garrison and Marth 2012)], and consensus sequence generation was performed with bcftools [v1.6 (Danecek et al. 2021)] and bedtools [v2.26.0 (Quinlan and Hall 2010)] by masking any positions with a sequencing depth of <10 reads.

Illumina reads were processed similarly as described above for MGI reads, with the following modifications: read quality control and trimming were performed with fastp [v0.20.0 (Chen et al. 2018a)]. Primers were trimmed using iVar [v1.3.1 (Grubaugh et al. 2019)], explicitly trimming primers from reads, and performing read remapping with bwa mem onto the reference genome afterward.

Successfully obtained SARS-CoV-2 genome sequences were uploaded to GISAID’s EpiCoV database alongside relevant metadata (Khare et al. 2021).

#### Phylogenetic analysis

All Latvian mink and farmworker SARS-CoV-2 isolate complete or near-complete genomes that were successfully reconstructed (*n* = 188 and *n* = 14, respectively) have been shared publicly previously and were retrieved from the GISAID EpiCoV database (Khare et al. 2021), along with the sequences of genotypes from the general population residing in the municipality where the farm is located that were collected during the period of surveillance at the farm (*n* = 59). These 261 sequences comprised the ‘core’ dataset analysed in the study. Additionally, the respective isolate sequence-derived features [e.g. Clade, Pango lineage (Rambaut et al. 2020), assigned protein amino-acid-sequence-altering substitutions, indel annotations, etc.] were downloaded in tabular form. GISAID uploaded sequence-associated metadata were verified with internal databases of the institutions involved in sample collection, processing, and sequencing. Only the isolate genomes unambiguously traceable internally in terms of metadata were selected to be used for subsequent analysis.

Selected sequences were then subjected to phylogenetic analysis using tools implemented in the augur package [v33.0.1 (Huddleston et al. 2021)]. Briefly, retrieved sequences were indexed, and sequences having ˃3000 ambiguous bases or missing data were filtered out. Filtered sequences (*N* = 261, available from GISAID under the following unique identifier: EPI_SET_250722ps) were then aligned to the Wuhan-Hu-1 SARS-CoV-2 reference genome (NC_045512.2) using the Multiple Alignment using Fast Fourier Transform (MAFFT) implementation [v7.526 (Katoh and Standley 2013)] distributed with augur, with gap-filling enabled. The resulting alignment was then used for maximum-likelihood tree reconstruction using IQ-TREE [v3.0.1 (Minh et al. 2020)] within augur (augur tree) using the GTR + F + I substitution model and allowing for polytomies, with the Wuhan-Hu-1 isolate set as an outgroup (other settings default). The resulting tree was annotated using the in-house processed Latvian SARS-CoV-2 sequence metadata database and Spike protein amino acid substitutions associated with the isolates as documented at GISAID (only for substitutions or deletions present in at least 10% of the individual mink isolate sequences from the dataset). Afterward, the resulting tree was refined using the ‘augur refine’ utility to produce a time-scaled tree [calculating confidence intervals for node dates, using marginal internal node date inference, opting for automatic determination of precision (selected as 2), retaining the Wuhan-Hu-1 isolate as a root, using ‘opt’ for ‘coalescent’ option, and setting clock filter at four interquartile ranges]. Ancestral traits/sequences were inferred based on the obtained tree using ‘augur traits’ and ‘augur ancestral’, with joint inference for the latter. Ancestral sequences inferred were then translated into the respective SARS-CoV-2 protein amino acid sequences (deriving annotations from NC_045512.2). The resulting tree, coupled with relevant metadata, was exported and interactively investigated using the auspice web server utilities associated with the Nextstrain framework (Hadfield et al. 2018), inputting the augur-pipeline-generated refined tree with inferred traits/sequence states at a given position. To increase the split dating precision within the main mink farm outbreak clade, given previous reports of the increased substitution rates of SARS-CoV-2 when crossing the human-to-mink species barrier, an additional refinement step was performed for the respective clade (Porter et al. 2023). The corresponding clade was pruned at its most recent common ancestor (MRCA) from the raw full tree, and subsequently used as an input for augur refine step, which was performed using the corresponding sequence subset from the initial full dataset multiple sequence alignment against Wuhan-Hu-1, with other analysis parameters remaining the same (however, this time keeping the root at the MRCA node at which the outbreak subtree was pruned from the full tree). No sequences were filtered out by the clock filter set at four interquartile ranges in either case. The resulting molecular clock parameters (substitution rate and its confidence interval, as well as *R*^2^) were documented from the results for both the full tree and the mink farm B.1.177.60 outbreak clade subtree. The mink farm B.1.177.60 outbreak clade clock was used for reporting the dates of MRCAs harbouring mutations of interest.

Annotated phylogeny visualizations were performed using custom R code based on ape (Didier 2021), ggtree (Yu 2020) and ggplot2 (Wickham 2009), ggnewscale (Campitelli 2025), dplyr (Wickham et al. 2025), phytools (Revell 2024), and treeio (Wang et al. 2020) library capabilities, with the resulting figure manually touched up in Inkscape (Inkscape Project 2020) whenever relevant.

Prior to conducting the analyses presented herein, the dataset investigated was assessed against the broader publicly available spatio-temporal context found in GISAID at the beginning of 2026, which has failed to provide additional sequences of immediate relevance for inclusion in the core dataset described in this work. First, an exploration of the global diversity of B.1.177.60 lineage sequences and their connection to the outbreak presented in this work was conducted. A larger-scale MAFFT alignment (Katoh and Standley 2013) and reconstruction of a maximum likelihood (ML) phylogeny [under the same settings as for our core dataset analysis (Minh et al. 2020)] were performed for a dataset of 4321 GISAID-downloaded SARS-CoV-2 isolate genomes sampled worldwide between 1 February 2021 and 1 March 2022 and belonging to the B.1.177.60 lineage. However, for this exploration, no quality cut-offs or any other filtering were performed to retrieve as many potentially relevant sequences as possible. The exploration confirmed that the mink B.1.177.60 sequences described here form a monophyletic clade not invaded by sequences from outside the country, and the outbreak was not fuelled by additional B.1.177.60 introductions. This failed to provide any additional recruitable sequences of high isolate genome quality and completely traceable metadata that might help in dissecting the outbreak described. For a finer investigation of Delta-derived (GK clade) mink sequences from the outbreak, relationships with sequences available globally were determined using Audacity/Instant functionality within GISAID on 10 May 2026 (Khare et al. 2021). The corresponding GK clade outbreak sequences were queried using a max distance of 5, a default quality threshold of 0.9, and considering up to 10 matches. Multiple sequence alignment, ML tree reconstruction, and subsequent tree refinement were performed via the augur framework (Huddleston et al. 2021), as previously mentioned for the core dataset.

## Results

### Surveillance results in minks and outbreak timeline

The first SARS-CoV-2-positive result was confirmed on 10 April 2021, in a dead mink. In total, 689 dead minks from the affected mink farm were tested during the surveillance period, and 269 of them were found virus-positive (RT-PCR-positive result). In total, only 30 (12.6%) oropharyngeal swabs taken from 238 live minks showed RT-PCR-positive results; all these RT-PCR-positive samples from live minks were collected in April, September, and October of the year 2021. Likewise, 238 blood samples taken from live minks were tested by ELISA, and 179 of them were found seropositive, as illustrated in Table 1. Samples from live minks for virological and serological testing were not taken in July and November 2021, and March 2022.

**Figure 1:**
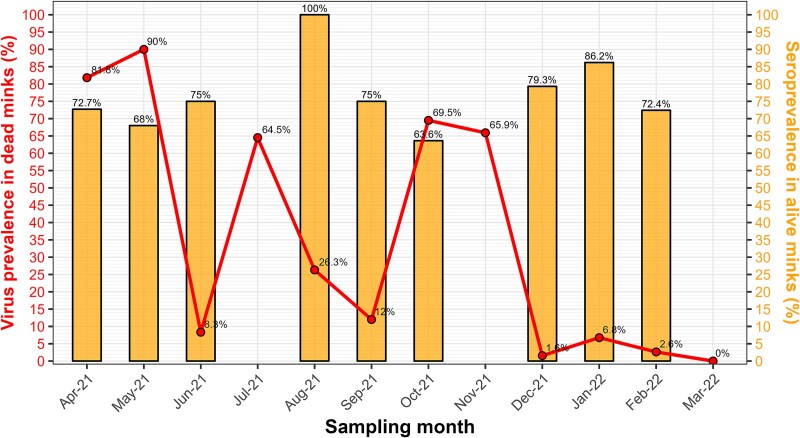
Monthly prevalence of SARS-CoV-2 at the affected mink farm throughout the surveillance period; red dots connected by a line represent the percentage of SARS-CoV-2 prevalence in dead minks as evaluated by RT-PCR; orange bars represent the percentage of seroprevalence in live minks; no testing of live minks was performed in July and November 2021, and March 2022.

The highest virus prevalence in dead minks was observed in April and May (81.8% and 90%, respectively), July (64.5%), October, and November of the year 2021 (69.5% and 65.9%, respectively), indicating three peaks during the surveillance period (Fig. 1). The virus prevalence reduced significantly in December 2021 after pelting season (November–December), when ˃85% of minks at the farm were pelted, leaving only breeding animals for the next season. The last virus-positive result in a dead mink was detected on 11 February 2022, while the last virus-positive result in a swab taken from a live mink was detected in a sample collected in October 2021. Restrictions set in place following the SARS-CoV-2 detection in the respective mink farm in April 2021 were lifted on 4 March 2022, following three consecutive weeks of absence of virus-positive results in samples collected from dead minks.

### Surveillance results in farm workers

**Figure 2:**
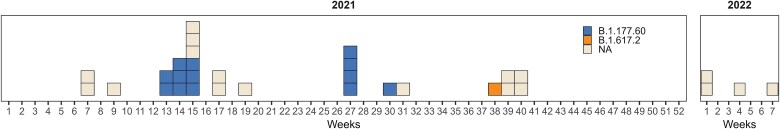
The number of weekly COVID-19 cases confirmed in mink farm workers from February 2021 to March 2022; squares represent individual cases, and square colouration indicates the respective lineage designation (according to the legend) for cases where the genome of the corresponding isolate could be successfully sequenced and reconstructed; ‘NA’ represents cases from which the corresponding SARS-CoV-2 genomes could not be successfully sequenced and reconstructed for inclusion in the analyses.

There were 77 farm workers associated with the mink farm. During the period from 17 February 2021 to 4 March 2022, a total of 32 COVID-19 cases were identified among farm workers aged 15–62 years (24 men and 8 women). The dynamics of COVID-19 cases in farm workers during the outbreak in the mink farm are presented in Fig. 2. None of the infected farm workers were required to be hospitalized. The first two COVID-19 cases were identified during the epidemiological investigation carried out after the confirmation of the outbreak in the mink farm (10 April 2021) and dated back to 17 February 2021 (the second case had been in contact with the first case at the mink farm). Therefore, these first two cases in farm workers are considered a potential cause of SARS-CoV-2 introduction to the mink farm. Sadly, material from neither of these earliest human cases could be successfully sequenced.

Fourteen farm workers (44%) with confirmed COVID-19 infection reported typical symptoms, while 18 (56%) had an asymptomatic course of illness, continued attending the workplace, and were detected through routine screening. Additionally, during the same period, three farm workers were diagnosed with COVID-19 after returning from abroad (these cases were not related to their stay in Latvia or work at the farm).

Out of 32 COVID-19 cases registered at the farm, four employees were fully vaccinated against COVID-19, and one had started but not completed the vaccination due to illness. The remaining individuals were unvaccinated.

The epidemiological investigation of the outbreak at the farm was initiated in April 2021. Starting from 12 April 2021, measures to limit the spread of infection were implemented, including strict isolation for infected farm workers and home quarantine for identified contacts.

Testing of farm workers was conducted based on epidemiological indications. In addition, routine weekly screening of farm workers was introduced on 12 April 2021 at the mink farm. As a result of routine screening, 21 COVID-19 cases were detected among farm workers, accounting for 66% of all cases at the farm.

Samples from 14 COVID-19-positive farm workers could be successfully sequenced and subjected to a complete or near-complete SARS-CoV-2 isolate genome reconstruction.

According to the results of the epidemiological investigation of COVID-19 cases, the possible introduction of SARS-CoV-2 into minks at the farm occurred from the first infected farm workers in February 2021, whose samples were not sequenced (Fig. 2).

### The outbreak was driven by the introduction of the B.1.177.60 SARS-CoV-2 lineage to the farm

SARS-CoV-2 RT-PCR-positive samples were subjected to the amplicon-based sequencing of the virus, allowing reconstruction of the associated (near-)complete SARS-CoV-2 genomes for 188 distinct animals and, in collaboration with the Latvian Centre for Disease Prevention and Control, 14 humans working at the farm throughout the surveillance period.

**Figure 3:**
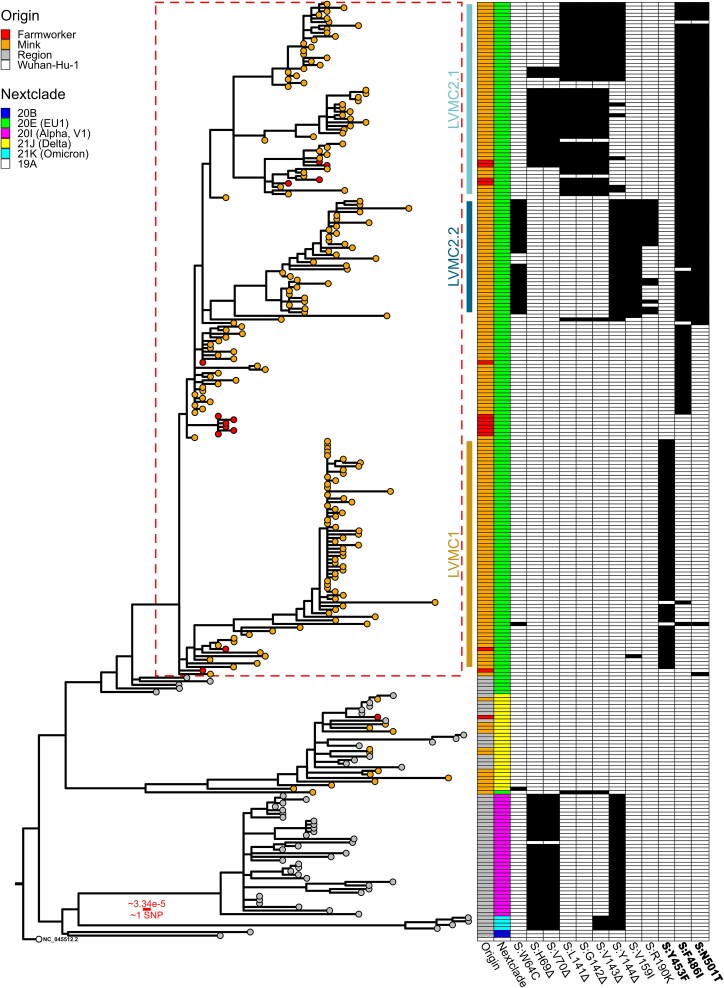
Genomic diversity of the successfully sequenced Latvian SARS-CoV-2 isolates connected to the respective mink farm; ML tree with 262 tips representing the obtained SARS-CoV-2 genomes of high-quality (>90% genome coverage) corresponding to the isolates originating from samples collected from minks (*n* = 188), mink farm workers (*n* = 14), and COVID-19-positive people from the respective municipality (*n* = 59), with sample collection dates spanning from 25 January 2021 to 21 January 2022 (first successfully sequenced positive farmworker and mink isolate collection dates are 1 and 8 April 2021, respectively) and the Wuhan-Hu-1 reference sequence (NC_045512.2) at the root; the sequences shown in the tree are annotated using the columns next to the respective leaves; the columns are coloured according to the legend; the first column shows the respective sample origin, the second—the respective SARS-CoV-2 isolate sequence clade; the remaining columns are coloured based on the presence of the respective SARS-CoV-2 spike protein amino acid change (black) or its absence (white) for changes that are associated with at least 10% (*n* ≥ 18) mink-derived isolate sequences; labels for changes in spike protein amino acid positions recognized for their role in mink-specific adaptation are highlighted in bold; the tree is drawn to scale, with the scale bar representing the distance corresponding to one nucleotide substitution; MAFFT-generated (v7.526) multiple sequence alignment (29 903 columns, 2594 distinct patterns, 314 parsimony-informative, 505 singleton sites, 29 084 constant sites) was used for ML phylogeny reconstruction under the GTR + F + I substitution model in IQ-TREE v3.0.1; the clade comprising sequences associated with a single B.1.177.60 lineage introduction is indicated by the dashed-line red rectangle; distinct co-circulating clades of closely-related isolates designated by us as **L**at**v**ian **M**ink **C**luster (LVMC) 1, 2.1, and 2.2 sequences are indicated by bars of different colours.

The genomic diversity and lineage designations from the whole dataset of the isolates successfully sequenced within the timeframe of the genomic surveillance suggest several independent introductions of SARS-CoV-2 to the largest Latvian mink farm (Fig. 3). However, the major outbreak of interest at the farm initially began with the introduction of a strain associated with the Nextclade clade 20E (EU1), more specifically, a PANGOLIN lineage B.1.177.60 designation (Rambaut et al. 2020, O’Toole et al. 2021), in the winter/early spring of 2021. Lineage B. 1.177.60-derived sequences accounted for ~93.06% of successfully sequenced isolates directly associated with the farm, 188 out of 202 associated isolates in total, 175/188 mink isolates, and 13/14 farm worker isolates (Fig. 3).

The earliest SARS-CoV-2 sequences associated with the farm corresponded to the aforementioned B.1.177.60 lineage and belonged to the farmworkers (collected on the first dates of April 2021). As of 22 June 2025, there were 5834 sequences of isolates belonging to this lineage available in the GISAID EpiCoV database (Khare et al. 2021). The first available sequence, designated as B.1.177.60, was derived from a sample with a collection date indicated as 17 August 2020, originating from Scotland. However, it was Northern Europe and the Baltic region where the lineage was documented more frequently, reaching cumulative prevalence as high as ~7% and ~3% among the isolates sequenced in Lithuania and Latvia, respectively, based on the given lineage ‘Outbreak.info’ genomic report for B.1.177.60 (Gangavarapu et al. 2023, Tsueng et al. 2023). The lineage in question is one of many derivatives of the B.1.177 ‘European’ lineage, also known as ‘20E (EU1)’ Nextstrain clade or ‘GV’ GISAID major clade. Based on the non-synonymous substitutions seen in at least 75% of the B.1.177.60 sequences globally, their most recent common ancestor did not harbour any particularly concerning mutations and had as few as eight amino-acid-altering mutations in total (ORF1b: P314L; S: L18F, A222V, D614G; ORF3a:V77F; ORF8:S84L; N:A220V; ORF10: V30L) (Gangavarapu et al. 2023, Tsueng et al. 2023).

**Figure 4:**
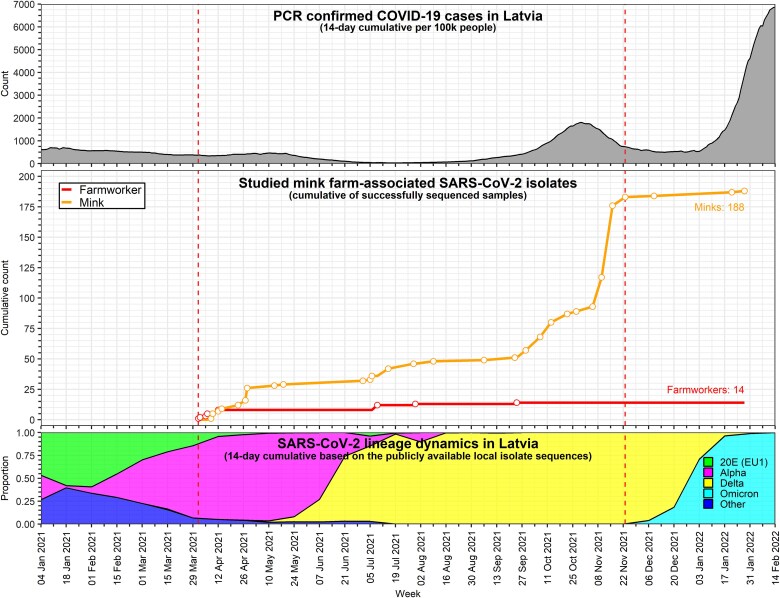
COVID-19 incidence (upper tile) and SARS-CoV-2 lineage dynamics (lower tile) in Latvia during the genomic surveillance period at the largest Latvian mink farm (middle tile); red dotted lines indicate collection dates of the samples from which the first (8 April 2021) and the last (23 November 2021) SARS-CoV-2 isolate sequences associated with a B.1.177.60 lineage introduction that drove the outbreak at the respective mink farm for a period of ˃8 out of 11 months were reconstructed.

Although B.1.177, along with its derivative lineages, was widespread in Europe in the second half of 2020, following the emergence of a more evolutionarily successful VOC Alpha (B.1.1.7), Alpha rapidly became the dominant lineage worldwide, leading to the gradual displacement of B.1.177 and B.1.177-derived lineages. The last known human sequences of the B.1.177.60 lineage were reported in Lithuania, with collection dates indicated as 11 November 2021. The same trend of B.1.177-derived lineage displacement holds true for the situation in Latvia; at the time of documentation of the first COVID-19-positive farmworker case in early April 2021, 20E (EU1) sequences (B.1.177.60 included) were identified in <20% of the sequenced cases in Latvia, with VOC Alpha isolates representing the majority of sequenced isolate genomes in the overall population. The B.1.177.60 lineage, however, became established at the largest Latvian mink farm, where it could further evolve in the farmed mink population while its related lineages were replaced by VOC Alpha (B.1.1.7), which was eventually displaced by VOC Delta (B.1.617.2) or its descendants (AY lineages), both in the Latvian and global human populations (Fig. 4, lower tile).

**Figure 5:**
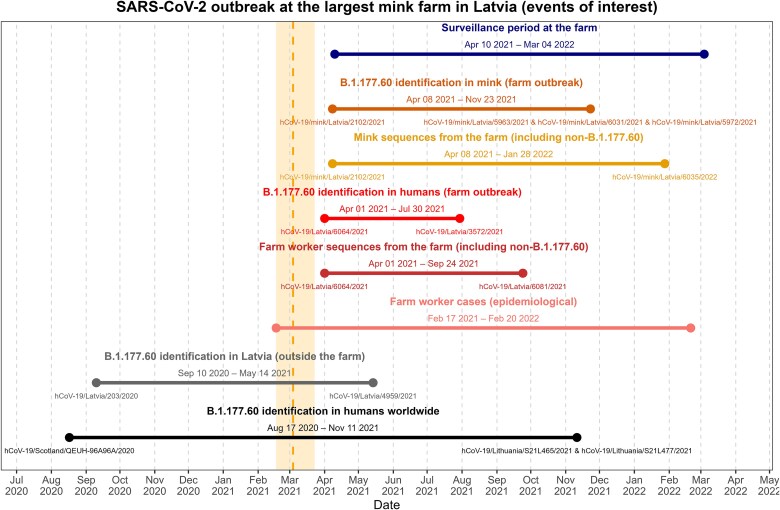
Timeline of the events of major interest regarding the described SARS-CoV-2 outbreak at the largest Latvian mink farm; date intervals for the corresponding events are given above the lines denoting them, and they are bounded by points representing the first and last instance; in the case of sequenced isolates, the boundaries of the intervals are labelled according to the corresponding isolate(s); the shaded orange area with a dashed line within it indicates the confidence interval and the inferred date of the MRCA responsible for initiation of the outbreak.

The initial introduction of B.1.177.60 soon led to the emergence and co-circulation of the mink-farm-restricted sister lineages that evolved and coexisted at the farm for nearly the whole period of the outbreak, allowing us to track their evolution in the novel host population *in situ* for a period of ˃8 months (Figs 3–5). The ancestral sequence reconstruction suggests that the virus genotype introduced to the farm also very likely had an ORF1b:G814F and an ORF3a:L219V amino acid change in addition to the ‘classical’ B.1.177.60 amino acid-altering mutations listed previously in this section. As the farm-associated sequences eventually formed well-defined monophyletic clades, we were able to denote at least three comparatively large **L**atvian **m**ink **f**arm SARS-CoV-2 isolate **c**lades (LVMC1, LVMC2.1, LVMC2.2; Fig. 3) and calculate that the likely introduction of the virus to the farm via the farm worker took place somewhere around 4 March 2021 as the inferred date (date confidence interval from 17 February to 23 March 2021).

During the surveillance period, animal-to-human transmission was documented in LVMC1 (one person affected) and LVMC2.1 (four persons affected) clades, in addition to one person being infected by the F486I-bearing B.1.177.60 genotype in early April. No human COVID-19 cases linked to the LVMC2.2 mink isolates were found during the surveillance period, despite this lineage making up a notable share of the farm-associated samples. Additionally, a cluster of six early farm worker cases consistent with a brief human-to-human transmission in early April 2021 was noted.

Fourteen sequences from the samples collected in relation to the outbreak at the farm during the period of 29 July 2021 (isolate under the name hCoV-19/mink/Latvia/3570/2021) to 28 January 2022 (isolate under the name hCoV-19/mink/Latvia/6035/2022) were found to belong to the GK clade associated with the Delta lineage. These included 13 sequences from minks and a single sequence from a farmworker. The aforementioned sequences did not form tight monophyletic clades comprising more than two sequences, but rather clustered together with epidemiologically unrelated (without any apparent association to the farm itself) isolates originating from the region where the farm is located. Notably, four Delta-derived isolate genomes could be obtained from mink samples collected in January 2022, whereas the last B.1.177.60 lineage descendants could be detected from samples collected on 23 November 2021 (Figs 3 and 5).

### Mutational landscape of the mink isolates and spike protein mutation emergence

Among the 188 mink isolates successfully sequenced, we have documented 433 different SARS-CoV-2 protein amino acid sequence-altering mutations (Supplementary Table S1). Only 55 (12.7%) such mutations were present in ˃10% of the successfully sequenced mink-derived genotypes, while 339 (~78.3%) were found in five or fewer isolates. Interestingly, 296 (~68.4%) mutations affecting amino acid sequences of the proteins were registered in no more than a single isolate.

**Figure 6:**
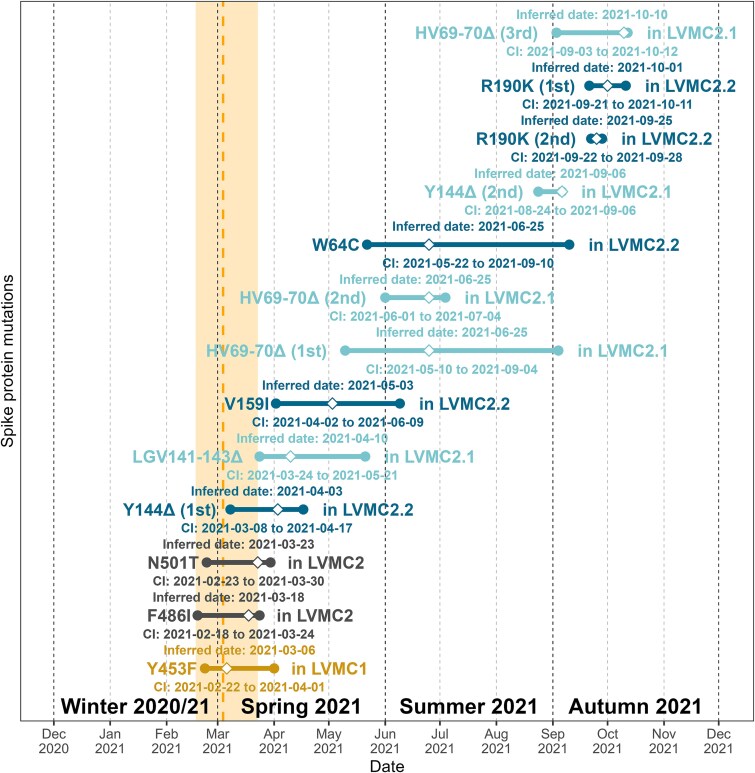
Inferred emergence time ranges for studied outbreak-associated viral isolates bearing mutations of interest that gave rise to Latvian mink farm isolate clusters that have co-circulated at the farm; the values represent individual emergence events and the inferred node information extracted from the time-scaled ML tree; the most likely inferred date associated with each event is represented by a white rectangle bordered by lollipops extending to the earliest and latest inferred confidence interval range dates; the *Y*-axis is categorical, and the individual events are arbitrarily coloured according to our designated clusters associated with the given outbreak for easier disambiguation; minor *X*-axis ticks are represented by grey dashed vertical lines separating months, and the major tick marks (black) separate seasons of the year; a semi-transparent orange box spans the inferred emergence date confidence interval for the isolate responsible for initiating the given outbreak, while the orange dashed vertical line corresponds to the most likely inferred date (4 March 2021); a subtree pruned at the outbreak MRCA from the full tree was used for date inference refinement to account for the difference in the SARS-CoV-2 evolutionary rate upon jumping into the mink population.

A closer look at the 55 mutations with >10% prevalence in 188 mink sequences from the farm reveals that 14 (~25.5%) such mutations were localized within the S protein coding gene, while 58 amino acid positions of the spike protein have been registered to differ from the Wuhan-Hu-1 reference sequence in total (Supplementary Table S1). Some of the most prevalent spike changes not associated with the founding isolate lineage included: W64C, HV69–70 deletion, LGV141–143 deletion, Y144 deletion, V159I, R190K, Y453F, F486I, and N501T. Moreover, convergent evolution of deletions affecting spike amino acid positions 69, 70, and 144 has been detected (Figs 3, 6, and  8). Additionally, reversions of the lineage B.1.177.60-associated mutations to the wild-type isolate Wuhan-Hu-1 base states were observed on multiple independent occasions (e.g. C21614T resulting in S:F18L, Supplementary Fig. S2).

Based on the augur refine analysis, the substitution rate of the whole tree was calculated as 7.167 × 10^−4^ ± 1.044 × 10^−4^ with a moderate temporal signal (*R*^2^ = 0.51). For the mink farm outbreak clade, the substitution rate was calculated as 8.300 × 10^−4^ ± 1.533 × 10^−4^ and demonstrated a better temporal signal (*R*^2^ = 0.75). Although there is a notable overlap of the uncertainty ranges, the outbreak clade subtree showed a better molecular clock signal and a slightly higher point estimate of the substitution rate in comparison to the full tree.

**Figure 7:**
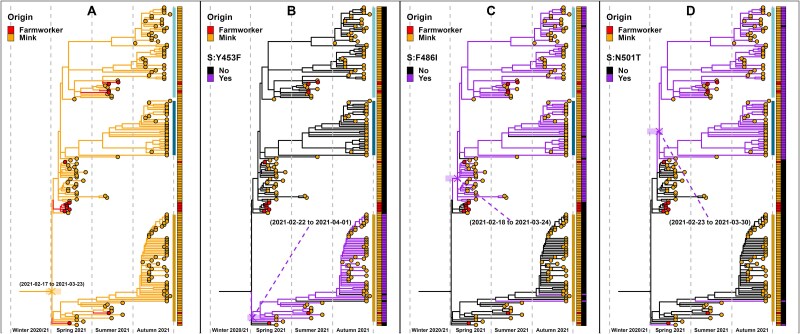
Time-scaled ML subtree from the MRCA of the B.1.177.60-associated sequences from the outbreak, annotated by sequence origin and spike protein mutations that have emerged and expanded the fastest; tile a shows the origin of the sequenced isolates, b—presence of the Y453F spike protein mutation, c—F486I spike protein mutation, and d—N501T spike protein mutation; in all of the trees, the tip colours represent the sequence origin (orange—minks, red—farmworkers); grey dashed lines delineate seasons of the year; predicted MRCA date confidence intervals for a particular trait are indicated as opaque bars at the respective nodes that are further indicated by the cross symbol; these nodes are also annotated with the predicted emergence date ranges (a—MRCA of the studied outbreak, b—S:Y453F MRCA, c—S:F486I MRCA, and d—S:N501T MRCA); the trees are further colour-coded according to their respective legends; coloured strips next to the tips indicate the ‘Latvian mink clusters’ as follows: LVMC1—orange, LVMC2.1—light blue, and LVMC2.2—dark blue.

Our data show that spike protein receptor-binding domain (RBD) amino acid-altering mutations resulting in Y453F and F486I, N501T were among the first ones to be selected for in viral isolates that established transmission among the minks at the farm after introduction of the B.1.177.60-derived MRCA between 17 February and 23 March 2021 (Figs 3, 6, and  7A–D and Supplementary Fig. S3).

**Figure 8:**
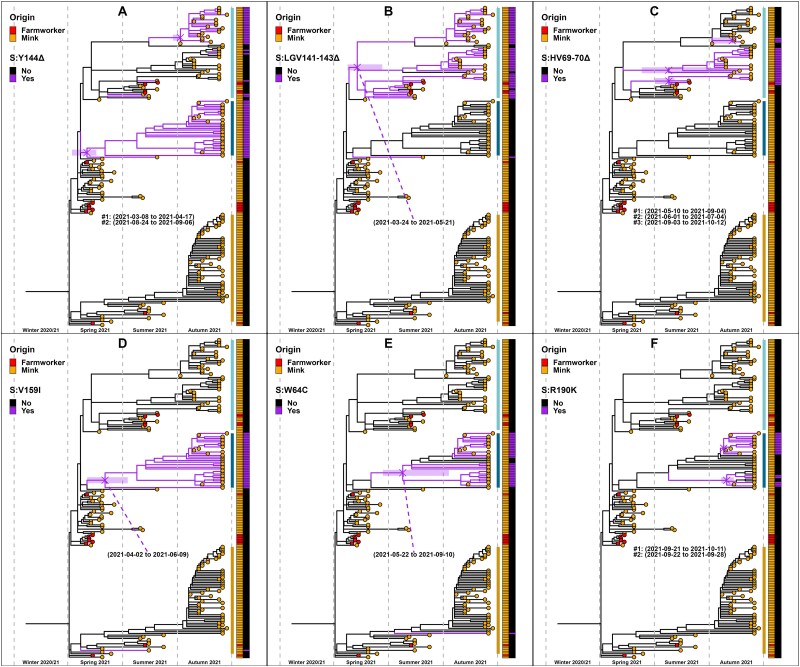
Time-scaled ML subtree from the MRCA of the B.1.177.60-associated sequences from the outbreak, annotated by selected traits demonstrating spike protein amino acid substitution differences between LVMC2.1 and LVMC2.2; tile a shows Y144 deletion emergence, b—LGV141–143 deletion, c—HV69–70 deletion, e—mutation resulting in V159I, e—mutation resulting in W64C, and f—mutation resulting in R190K; in all of the trees, the tip colours represent the sequence origin (orange—minks, red—farmworkers); grey dashed lines delineate seasons of the year; predicted MRCA date confidence intervals for a particular trait are indicated as opaque bars at the respective nodes that are further indicated by the cross symbol, these nodes are also annotated with the predicted emergence date ranges (a—MRCAs of the convergent S:Y144Δ, b—S:LGV141–143Δ MRCA, c—convergent S:HV69–70Δ MRCAs, d—S:V159I MRCA, e—S:W64C MRCA, and f—S:R190K MRCA); the trees are further colour-coded according to their respective legends; coloured strips next to the tips indicate the ‘Latvian mink clusters’ as follows: LVMC1—orange, LVMC2.1—light blue, and LVMC2.2—dark blue.

While the spike protein amino acid sequences of LVMC1 isolates did not seem to have acquired further expansive changes since the early emergence of S:Y453F, it was not the case for LVMC2.1 and LVMC2.2 ancestral isolates, which share an S:F486I and S:N501T-bearing MRCA (Supplementary Fig. S3). In about half a year, the LVMC2.2 lineage has managed to further acquire Y144 deletion (which happened independently in LVMC2.1 and 2.2; Figs 3 and 8A), V159I (Fig. 8D), W64C (Fig. 8E), and R190K (Fig. 8F) spike amino acid substitutions, whereas the LVMC2.1 lineage additionally evolved several other deletions (LGV141–143 and HV69–70 deletions), before acquiring a Y144 deletion, as well (Fig. 8A–C).

## Discussion

SARS-CoV-2 virus has been detected in 400 mink farms for fur production in eight countries in the European Union/European Economic Area (EU/EEA), namely 290 farms in Denmark, 1 in France, 21 in Greece, 1 in Italy, 2 in Lithuania, 69 in the Netherlands, 3 in Spain, and 13 in Sweden (European Food Safety Authority and European Centre for Disease Prevention and Control *et al*. 2021), and in Canada and the USA (Food and Agriculture Organization of the United Nations, World Organisation for Animal Health, and World Health Organization 2021). On one of the mink farms in Denmark, the presence of the virus was detected when the prevalence was low (only in 13% of tested mink) and with only 3% seroprevalence in the sampled animals, but the virus prevalence had increased to ~90% only 4 days later, and the seroprevalence had reached 97% after a further 8 days. This indicates a very rapid spread of the virus among the mink that were living in close proximity (Rasmussen et al. 2024). This corresponds to our surveillance results in the infected mink farm that revealed high seroprevalence (63.6%–100%) during the whole surveillance period. Our results of virus prevalence in dead mink also correspond to the Denmark study; we observed fluctuation of virus prevalence during the surveillance period, showing three peaks (in April and May, July, then October and November 2021). Surprisingly, only 30 (12.6%) oropharyngeal swabs taken from 238 live minks showed RT-PCR-positive results, showing a large difference with results obtained from dead minks. Quality of samples could be one of the reasons, but since all live minks were sampled by the same person and there were positive results found in April, September, and October, most probably such results are not caused by sampling errors. As the same live minks were tested both for the presence of virus genome and antibodies, and a high proportion of them were already seropositive, it might be that the virus genome was not present anymore in minks showing seropositive results. However, there were also minks that gave negative results both by PCR and serology. Still, a clear explanation is missing for why virus prevalence in live randomly selected minks was so low compared to dead minks. High seroprevalence rates, however, did not prevent minks from infections caused by the introduction of other SARS-CoV-2 virus strains but possibly played a role in reducing further spread of these virus strains among minks within a farm.

While infections in farmed mink were initially detected by observing severe respiratory symptoms and increased mortality, many infections were subclinical with no apparent clinical signs, making mink hidden animal reservoirs/sources for SARS-CoV-2 (Jahid et al. 2024). Also, in our study, increased morbidity and mortality in the infected mink farm were not observed during the whole surveillance period. This highlights the role of active surveillance, both virological and serological, in following the dynamics of this infection at the mink farm. Data obtained during our study by monitoring the dynamics of SARS-CoV-2 virus prevalence and seroprevalence in the infected mink farm for 11 months provide unique results to further advance the understanding of COVID-19 epidemiology in animals.

In addition to human-to-mink spillovers, anthropozoonotic spillovers from animals to humans were also detected and documented, as seen in the Netherlands and Denmark, including transmission between farms (Larsen and Paludan 2020, Munnink et al. 2021). In Latvia, 32 human cases were confirmed in farm workers during the surveillance period of 11 months at the affected mink farm. Despite different biosafety measures applied to the mink farm, including preventive measures for farm workers, it seems that under the circumstances of the COVID-19 pandemic situation, it is difficult to ensure full protection of humans working on mink farms and prevent virus spread to and from minks.

Following the detection of COVID-19 on mink farms, countries responded in different ways: some nations, like Denmark and the Netherlands, performed mass culling of entire mink farms due to concerns of spillover back into the human population, while others, including Canada and Lithuania, tried to contain the transmissions through testing, isolation, and disinfection (Jahid et al. 2024). In Poland, the culling of all animals on a farm was foreseen if the mink mortality rate exceeded 10% or in case of mink-to-human transmission. As this was not the case on the three virus-positive farms, culling was not performed (Domańska-Blicharz et al. 2023).

The presence of SARS-CoV-2 in samples from farmed minks in Latvia was first confirmed on 10 April 2021, while the first cases of COVID-19 in humans working at the same farm were traceable back to samples collected in February 2021. Considering the previous reports on SARS-CoV-2 evolution in the mink farming environments elsewhere in the world that have demonstrated the emergence of concerning mutations, this has spurred increased attention to mink farming in Latvia and has prompted extension of the surveillance efforts, which were already in place, with a follow-up genomic scrutiny of the samples.

In Latvia, immediately after the confirmation of SARS-CoV-2 infection at the mink farm, a cross sectional working group was established comprising the FVS, Centre for Disease Prevention and Control, Ministry of Agriculture, Ministry of Health, and invited experts and scientists from the Latvian Biomedical Research and Study Centre, the Institute of Food Safety, Animal Health and Environment (BIOR), and the Faculty of Veterinary Medicine of the Latvian University of Agriculture. *Ad hoc* meetings were organized on a regular basis (almost weekly), thus providing regular updates on the epidemiological situation both in animal and human populations, coordinated solutions regarding necessary safeguards and biosecurity measures, and implementation of the surveillance and diagnostic schemes in the mink farm and for the farm workers.

Continuous surveillance of the outbreak for a period of 11 months reveals the extensive circulation of the virus among the minks, resulting in an unhindered evolution of a single virus genotype derived from the B.1.177.60 lineage, which seems to have entered the mink farm through a single introduction event. The determined timing of this introduction seems to somewhat coincide with both the results of the traditional epidemiological investigation pointing to the second half of February, and the molecular clock analysis, which suggested the most likely point estimate for the inferred outbreak MRCA date as 4 March (date confidence interval from 17 February to 23 March 2021). Transmission of the isolates descended from the founder genotype introduced has continued for at least 9 months, resulting in an ability to document a notable degree of phylogenetic diversity of its descendants. To our knowledge, the data presented within this work represent the longest continuous single-introduction-driven *in situ* evolution of SARS-CoV-2 within a mink farm.

In early April 2021 (the first cases of positive farmworkers and minks at the said farm that were sequenced shared the same evolutionary descent and belonged to the same B.1.177.60 lineage), most of the random SARS-CoV-2 isolates sequenced from the global population in Latvia already belonged to the Alpha lineage, and the outbreak at the farm has continued throughout the Delta and into the Omicron dominance in the global human population. While no Alpha or Omicron variant sequences were obtained from the samples linked to this farm, Delta variant isolates could be reconstructed from several of the later mink samples (Supplementary Table S2). Interestingly, despite Delta also being introduced into minks kept at the farm, it did not manage to expand and overthrow the B.1.177.60 derivatives already established there for some time, judging from the dating of their most recent common ancestor. Based on the genotype diversity and clustering pattern of the Delta-derived isolate sequences we obtained from minks within the context of Delta isolates from humans inhabiting the region where the farm is located, a number of independent introductions of Delta-derived genotypes can be assumed. Given a relatively ample sampling throughout the outbreak at the farm, in case of notable transmission as a result of a single introduction, we would definitely expect to see relatively tight and distinct monophyletic Delta-derived isolate clusters comprising multiple sequences from mink material collected at different dates, which was not the case.

To further investigate non-B.177.60 isolates associated with the farm and assess the potential number of independent introductions, we have attempted to evaluate their branching within a broader context of the most closely-related sequences available globally at the date (Supplementary Fig. S4 and Supplementary Table S3). The reconstructed tree further validated that most of the GK clade sequences to be unrelated to each other at a level that could be suggestive of a long within-farm circulation of Delta derivatives, with most of the GK sequences originating from the farm still likely representing individual introductions. Notably, in the expanded context, only half of the sequences demonstrated placement within clades comprising either exclusively or mainly human isolates from Latvia. Some of the mink isolate sequences were still located at the tips of deep isolated branches, indicative of a lack of close sequenced relatives available publicly. However, interestingly, in one instance, a placement of a mink isolate within a clade comprising sequences of human origin from Lithuania was noted, whereas in another instance, one of the mink isolates shared an MRCA only with a single Latvian human isolate, both of which sat within a clade comprising 25 human isolates from Ireland.

While the introduction of other lineages to the farm (e.g. Alpha) during the surveillance period cannot be excluded, these were not detected based on the molecular investigations of the farm-associated samples using sequencing, and remain just a hypothetical possibility.

The reason behind the apparent inability of later variants that were dominant in the global human population to establish themselves and supersede B.1.177.60 descendants reigning supreme at the farm may lie in a rather quick selection of mutations adapting the virus specifically to minks when entering a substantially large and easily accessible population of initially immunologically naïve mink hosts. This means that nearly any of the other genotypes introduced later on from humans are figuratively ‘bringing a knife to a gunfight’, likely beginning the competition against a serious challenge at a great disadvantage. And while mutations in all genes found across the SARS-CoV-2 genome were documented throughout the outbreak, of special interest in light of the global pandemic response were the mutations resulting in changes of the SARS-CoV-2 spike protein amino acid sequence absent in the first sequenced isolates of the virus that have driven the pandemic, due to potential implications of such changes in both the cognate receptor binding affinity and immune evasion capabilities of the virus (Harvey et al. 2021).

In this regard, during the period of surveillance, the introduced isolate, expanding and having the descendants independently co-evolving at the farm, progressively acquired spike-protein-altering changes, many of which became fixed in the population, reached high frequency, and were detectable by the end of the outbreak fuelled by B.1.177.60 introduction (Fig. 4 and Supplementary Fig. S3). Interestingly, and somewhat surprisingly, while LVMC2 descendants were steadily accumulating such changes, many of which took off to expand, a less numerous LVMC1 clade did not show the appearance and fixation of any other change in the spike amino acid sequence further than S:Y453F. Thus, the B.1.177.60 genotype introduced to the farm has rapidly changed spike protein tyrosine at position 453 to phenylalanine to give rise to LVMC1 (Figs 3 and 7B). This coincides with the previous reports that Y453F, which arose convergently on multiple occasions in both farmed (Hammer et al. 2021, Munnink et al. 2021) and experimental (Everett et al. 2021) animal settings, significantly enhances the interaction of the virus with mink and other *Mustela* angiotensin-converting enzyme 2 (ACE2) orthologs, and, thus, adapts the virus to a new host (Ren et al. 2021).

The MRCA of the isolates belonging to LVMC2.1 and LVMC2.2, and some older isolates (without a distinct farm-associated clade designation by us), has acquired isoleucine in place of phenylalanine at spike position 486 almost at the same time as Y453F happened in clade LVMC1 (Figs 3 and 7C). This substitution contrasts with the more usual mink adaptation seen in this position on multiple occasions elsewhere (F486L) (Lu et al. 2021), as in our case, the phenylalanine at position 486 has changed to isoleucine (F486I). Notably, this mutation is also seen in two of the LVMC1 sequences not associated with the large F486I-bearing viral sequence clade, which had later split into LVMC2.1 and LVMC2.2 sequences, and the MRCA of which had acquired it early on after the introduction to the farm (Figs 3 and 7C). Although the exact implications of such a mutation have not been studied in detail, to our knowledge, it can be cautiously speculated that it might be adaptive and analogous to F486L to an extent. As for the epidemiologic consequences, this particular change was initially shown to be associated with neutralization failure by at least some neutralizing antibodies, and subsequently reaffirmed as offering immune evasion on other occasions (Francino-Urdaniz et al. 2021, Starr et al. 2021, Wilkinson et al. 2022, Liu et al. 2025).

According to our data, the mink-associated spike N501T (Richard et al. 2020, Eckstrand et al. 2021, Zhou et al. 2022) change seems to have arisen at least three times independently at the farm. However, only a single F486I + N501T-bearing genotype that emerged early during the period of surveillance among minks at the farm has had the opportunity to spread further, giving rise to LVMC2.1 and LVMC2.2 (Figs 3 and 7D). The wild-type spike protein amino acid at position 501 (Asn) was previously outlined as suboptimal (Wan et al. 2020) for binding human ACE2, with the N501T substitution being shown to improve binding affinity to human ACE2 (Starr et al. 2020). Thus, while being adaptive to minks, it is not surprising that this change has also previously occurred in lineages circulating among humans (Cai and Cai 2021). The N501T was also shown to have a higher ACE2 affinity and escape at least two neutralizing antibodies (Francino-Urdaniz et al. 2021).

The S:HV69 deletion was previously identified as a ‘recurrent deletion region’ based on the publicly available SARS-CoV-2 isolates (McCarthy et al. 2021), and has been documented in an immunosuppressed chronically infected individual treated with rituximab (in which a S:Y453F variant has emerged as well) (Bazykin et al. 2021). Another report of a deletion arising in an immunocompromised chronically infected patient documented the occurrence of S:LGVY141–144 deletion (Avanzato et al. 2020). The S:Y144 deletion, however, has also been shown to be associated with some antibody escape without being a part of the four-amino-acid deletion at positions 141–144 (Haynes et al. 2021, McCallum et al. 2021). At the same time, we were not able to find any reports that have functionally studied the impacts of V159I, W64C, and R190K spike mutations on either the binding efficiency changes to the cognate ACE2 receptor on the cells of the host, or any other properties of the SARS-CoV-2 (all three of the aforementioned mutations appeared as LVMC2.2 evolved at the farm under surveillance, Fig. 8D–F).

Although in this article we have focused on the evolution of spike protein amino acid sequence during the 11-month-long SARS-CoV-2 outbreak initiated at the largest Latvian mink farm by the lineage B.1.177.60 representative in the spring of 2021, potentially beneficial adaptations are obviously not restricted to the spike protein. It is believable that non-synonymous changes in other protein-coding nucleotide sequences could also play a role that is less evident, but advantageous to the virus in one way or the other, nevertheless. Thus, meta-analyses of the particular animal species-associated SARS-CoV-2 sequences might uncover other potentially adaptive hallmark mutations in other protein-coding sequences and initiate research on their phenotypical implications, expanding the repertoire of the sequence positions of interest.

Results of the genomic surveillance efforts at the human and animal interface that are still taking place worldwide are expected to add to the body of empirical knowledge that should prove helpful and be actionable in case of future novel coronavirus outbreaks.

In any case, the ‘Latvian mink genotypes’ seem not to have expanded beyond the given farm at which the outbreak occurred in an epidemiologically relevant manner. Based on the continuous collaborative Latvian SARS-CoV-2 genomic surveillance results encompassing testing of positive cases in the region and, later on, wastewater surveillance efforts within the country (Dejus et al. 2023), the mink-farm-associated genotypes were not detected in the local human population, likely due to the fact that the given farm is located in a remote area of a comparatively sparsely populated municipality, and the opportunities for social recreational activities, as well as human mobility generally, were limited at the time of the outbreak.

The data presented in this work highlight once more that the usual mink farming environment, in which hosting caged animals (‘susceptible hosts’ in virological parlance) in very close proximity to each other for prolonged periods of time is common, readily supports the transmission of SARS-CoV-2 among the mink populations. In addition to conditions favourable for animal-to-animal transmission, many cases of human-to-animal and animal-to-human transmission were recorded globally in such mink farming environments as well, regardless of the efforts to safeguard the people working at the mink–human interface. Taken together, the evidence from Latvia and elsewhere suggests that mink farming can create a risk for the establishment of virus reservoirs in which the founder viral lineages can freely explore their sequence space and accumulate adaptive mutations before re-entering the human population via farmworkers. Although one would expect that the virus should primarily become adapted to mink, genomic surveillance of such outbreaks remains essential to track the emergence of potentially concerning mutations, including novel combinations that could confer an advantage in populations with rising immunity to earlier SARS-CoV-2 variants.

This case demonstrated that Latvia provides a strong example of effective crisis management through the co-ordinated application of the One Health approach. The response was implemented step by step, with continuous situation monitoring, systematic risk assessment, and evidence-based decision-making. Regular data analysis, risk assessment, and systematic surveillance were essential elements in ensuring an effective and timely response during the outbreak. These findings highlight the critical value of co-ordinated surveillance within the One Health framework, emphasizing the necessity and significance of close collaboration between veterinary and public health authorities.

## Supplementary Material

Supplementary_materials_veag038

## Data Availability

The core dataset sequences analysed in this study are available from the GISAID EpiCoV database, under the following unique sequence set identifier (EPI_SET ID): EPI_SET_250722ps. The dataset of the farm-associated GK clade sequences and their closest relatives found globally is available from the GISAID EpiCoV database, under the following unique sequence set identifier (EPI_SET ID): EPI_SET_260510vs. A subset of isolate sequences generated during this study from mink and farmworker samples that successfully passed the automated GenBank SARS-CoV-2 submission quality-control procedures is also available from GenBank under accession numbers PZ374406–PZ374584.

## References

[ref1] Avanzato VA, Matson MJ, Seifert SN et al. Case study: prolonged infectious SARS-CoV-2 shedding from an asymptomatic immunocompromised individual with cancer. *Cell* published online 2020;183:1901–12.e9. 10.1016/j.cell.2020.10.049 33248470 PMC7640888

[ref2] Bashor L, Gagne RB, Bosco-Lauth AM et al. SARS-CoV-2 evolution in animals suggests mechanisms for rapid variant selection. *Proc Natl Acad Sci U S A* 2021;118:1–10. 10.1073/pnas.2105253118 PMC8612357 34716263

[ref3] Bazykin G, Stanevich O, Danilenko D et al. Emergence of Y453F and Δ69–70HV mutations in a lymphoma patient with long-term COVID-19. 2021. Virological.org. https://virological.org/t/emergence-of-y453f-and-69-70hv-mutations-in-a-lymphoma-patient-with-long-term-covid-19/580 (30 June 2022, date last accessed).

[ref4] Cai HY, Cai A. SARS-CoV2 spike protein gene variants with N501T and G142D mutation-dominated infections in mink in the United States. *J Vet Diagn Invest* 2021;33:939–42. 10.1177/10406387211023481 34109885 PMC8366104

[ref5] Campitelli E . ggnewscale: Multiple Fill and Color Scales in ggplot2. Zenodo, 2025. 10.5281/zenodo.15702781

[ref6] Chen S, Zhou Y, Chen Y et al. Fastp: an ultra-fast all-in-one FASTQ preprocessor. *Bioinformatics* 2018a;34:i884–90. 10.1093/bioinformatics/bty560 30423086 PMC6129281

[ref7] Chen Y, Chen Y, Shi C et al. SOAPnuke: a MapReduce acceleration-supported software for integrated quality control and preprocessing of high-throughput sequencing data. *Gigascience* 2018b;7:1–6. 10.1093/gigascience/gix120 PMC5788068 29220494

[ref8] Crits-Christoph A, Levy JI, Pekar JE et al. Genetic tracing of market wildlife and viruses at the epicenter of the COVID-19 pandemic. *Cell* 2024;187:5468–82.e11. 10.1016/j.cell.2024.08.010 39303692 PMC11427129

[ref9] Danecek P, Bonfield JK, Liddle J et al. Twelve years of SAMtools and BCFtools. *Gigascience* 2021;10:giab008. 10.1093/gigascience/giab008 33590861 PMC7931819

[ref10] De Wit E, Van Doremalen N, Falzarano D et al. SARS and MERS: recent insights into emerging coronaviruses. *Nat Rev Microbiol* 2016;14:523–34. 10.1038/nrmicro.2016.81 27344959 PMC7097822

[ref11] Dejus B, Cacivkins P, Gudra D et al. Wastewater-based prediction of COVID-19 cases using a random forest algorithm with strain prevalence data: a case study of five municipalities in Latvia. *Sci Total Environ* 2023;891:164519. 10.1016/j.scitotenv.2023.164519 37268136 PMC10229444

[ref12] Didier G . Package ‘Ape’*,* 2021. https://cran.r-project.org/web/packages/ape/ape.pdf.

[ref13] Domańska-Blicharz K, Munnink BBO, Orłowska A et al. Cryptic SARS-CoV-2 lineage identified on two mink farms as a possible result of long-term undetected circulation in an unknown animal reservoir, Poland, November 2022 to January 2023. *Euro Surveill* 2023;28:2300188. 10.2807/1560-7917.ES.2023.28.16.2300188 37078885 PMC10283451

[ref14] Drexler JF, Corman VM, Drosten C. Ecology, evolution and classification of bat coronaviruses in the aftermath of SARS. *Antivir Res* 2014;101:45–56. 10.1016/j.antiviral.2013.10.013 24184128 PMC7113851

[ref1c] Corman VM, Landt O, Kaiser M et al. Detection of 2019 novel coronavirus (2019-nCoV) by real-time RT-PCR. Euro surveillance: bulletin Europeen sur les maladies transmissibles = European communicable disease bulletin, 2020;25:2000045. 10.2807/1560-7917.ES.2020.25.3.2000045 PMC6988269 31992387

[ref15] Eckstrand CD, Baldwin TJ, Rood KA et al. An outbreak of SARS-CoV-2 with high mortality in mink (*Neovison vison*) on multiple Utah farms. *PLoS Pathog* 2021;17:e1009952. 10.1371/journal.ppat.1009952 34767598 PMC8589170

[ref16] European Food Safety Authority and European Centre for Disease Prevention and Control, Boklund A, Gortázar C et al. Monitoring of SARS-CoV-2 infection in mustelids. *EFSA J* 2021;19:e06459. 10.2903/j.efsa.2021.6459 33717355 PMC7926496

[ref17] Everett HE, Lean FZX, Byrne AMP et al. Intranasal infection of ferrets with SARS-CoV-2 as a model for asymptomatic human infection. *Viruses* 2021;13:1–14. 10.3390/v13010113 PMC7830262 33467732

[ref18] Food and Agriculture Organization of the United Nations, World Organisation for Animal Health, World Health Organization . *SARS-CoV-2 in Animals Used for Fur Farming: GLEWS + Risk Assessment, 20 January 2021.* 2021. https://www.who.int/publications/i/item/WHO-2019-nCoV-fur-farming-risk-assessment-2021.1 (6 February 2026, date last accessed).

[ref19] Francino-Urdaniz IM, Steiner PJ, Kirby MB et al. One-shot identification of SARS-CoV-2 S RBD escape mutants using yeast screening. *Cell Rep* 2021;36:109627. 10.1016/j.celrep.2021.109627 34416153 PMC8352667

[ref20] Gangavarapu K, Latif AA, Mullen JL et al. Outbreak.info genomic reports: scalable and dynamic surveillance of SARS-CoV-2 variants and mutations. *Nat Methods* 2023;20:512–22. 10.1038/s41592-023-01769-3 36823332 PMC10399614

[ref21] Garrison E, Marth G. Haplotype-based variant detection from short-read sequencing. arXiv:1207.3907. Preprint, arXiv, 2012. 10.48550/arXiv.1207.3907

[ref22] Grubaugh ND, Gangavarapu K, Quick J et al. An amplicon-based sequencing framework for accurately measuring intrahost virus diversity using PrimalSeq and iVar. *Genome Biol* 2019;20:8. 10.1186/s13059-018-1618-7 30621750 PMC6325816

[ref23] Hadfield J, Megill C, Bell SM et al. NextStrain: real-time tracking of pathogen evolution. *Bioinformatics* 2018;34:4121–3. 10.1093/bioinformatics/bty407 29790939 PMC6247931

[ref24] Hale VL, Dennis PM, McBride DS et al. SARS-CoV-2 infection in free-ranging white-tailed deer. *Nature* 2022;602:481–6. 10.1038/s41586-021-04353-x 34942632 PMC8857059

[ref25] Hammer AS, Quaade ML, Rasmussen TB et al. SARS-CoV-2 transmission between mink (*Neovison vison*) and humans, Denmark. *Emerg Infect Dis* 2021;27:547–51. 10.3201/eid2702.203794 33207152 PMC7853580

[ref26] Harvey WT, Carabelli AM, Jackson B et al. SARS-CoV-2 variants, spike mutations and immune escape. *Nat Rev Microbiol* 2021;19:409–24. 10.1038/s41579-021-00573-0 34075212 PMC8167834

[ref27] Haynes WA, Kamath K, Lucas C et al. Impact of B.1.1.7 variant mutations on antibody recognition of linear SARS-CoV-2 epitopes. medRxiv*,* 2021.

[ref28] Hobbs EC, Reid TJ. Animals and SARS-CoV-2: species susceptibility and viral transmission in experimental and natural conditions, and the potential implications for community transmission. *Transbound Emerg Dis* published online 2021;68:1850–67. 10.1111/tbed.13885 33091230 PMC8359434

[ref29] Holmes EC, Goldstein SA, Rasmussen AL et al. The origins of SARS-CoV-2: a critical review. *Cell* 2021;184:4848–56. 10.1016/j.cell.2021.08.017 34480864 PMC8373617

[ref30] Huddleston J, Hadfield J, Sibley TR et al. Augur: a bioinformatics toolkit for phylogenetic analyses of human pathogens. *J Open Source Softw* 2021;6:2906. 10.21105/joss.02906 34189396 PMC8237802

[ref31] Inkscape Project . Inkscape. 2020. https://inkscape.org (10 May 2021, date last accessed.

[ref32] Jahid MJ, Bowman AS, Nolting JM. SARS-CoV-2 outbreaks on mink farms—a review of current knowledge on virus infection, spread, spillover, and containment. *Viruses* 2024;16:81. 10.3390/v16010081 38257781 PMC10819236

[ref33] Katoh K, Standley DM. MAFFT multiple sequence alignment software version 7: improvements in performance and usability. *Mol Biol Evol* published online 2013;30:772–80. 10.1093/molbev/mst010 23329690 PMC3603318

[ref34] Khare S, Gurry C, Freitas L et al. GISAID’s role in pandemic response. *China CDC Wkly* 2021;3:1049–51. 10.46234/ccdcw2021.255 34934514 PMC8668406

[ref35] Larsen CS, Paludan SR. Corona’s new coat: SARS-CoV-2 in Danish minks and implications for travel medicine. *Travel Med Infect Dis* 2020;38:101922. 10.1016/j.tmaid.2020.101922 33227502 PMC7678454

[ref36] Li H . Aligning sequence reads, clone sequences and assembly contigs with BWA-MEM. arXiv:1303.3997. Preprint, arXiv, 2013. 10.48550/arXiv.1303.3997

[ref37] Liu W, Huang Z, Xiao J et al. Evolution of the SARS-CoV-2 omicron variants: genetic impact on viral fitness. *Viruses* 2024a;16:184. 10.3390/v16020184 38399960 PMC10893260

[ref38] Liu WJ, Liu P, Lei W et al. Surveillance of SARS-CoV-2 at the Huanan Seafood Market. *Nature* 2024b;631:402–8. 10.1038/s41586-023-06043-2 37019149

[ref39] Liu X, Nie Z, Si H et al. Generative prediction of real-world prevalent SARS-CoV-2 mutation with *in silico* virus evolution. *Brief Bioinform* 2025;26:bbaf276. 10.1093/bib/bbaf276 40532108 PMC12204194

[ref40] Lu L, Sikkema RS, Velkers FC et al. Adaptation, spread and transmission of SARS-CoV-2 in farmed minks and associated humans in the Netherlands. *Nat Commun* 2021;12:1–12. 10.1038/s41467-021-27096-9 34815406 PMC8611045

[ref41] McCallum M, De Marco A, Lempp FA et al. N-terminal domain antigenic mapping reveals a site of vulnerability for SARS-CoV-2. *Cell* published online 2021;184:2332–47.e16. 10.1016/j.cell.2021.03.028 33761326 PMC7962585

[ref42] McCarthy KR, Rennick LJ, Nambulli S et al. Recurrent deletions in the SARS-CoV-2 spike glycoprotein drive antibody escape. *Science* published online 2021;371:1139–42. 10.1126/science.abf6950 33536258 PMC7971772

[ref43] Minh BQ, Schmidt HA, Chernomor O et al. IQ-TREE 2: new models and efficient methods for phylogenetic inference in the genomic era. *Mol Biol Evol* 2020;37:1530–4. 10.1093/molbev/msaa015 32011700 PMC7182206

[ref44] Molenaar RJ, Vreman S, Hakze-van der Honing RW et al. Clinical and pathological findings in SARS-CoV-2 disease outbreaks in farmed mink (*Neovison vison*). *Vet Pathol* 2020;57:653–7. 10.1177/0300985820943535 32663073

[ref45] Moreno A, Lelli D, Trogu T et al. SARS-CoV-2 in a mink farm in Italy: case description, molecular and serological diagnosis by comparing different tests. *Viruses* 2022;14:1738. 10.3390/v14081738 36016360 PMC9415545

[ref46] Munnink BBO, Sikkema RS, Nieuwenhuijse DF et al. Transmission of SARS-CoV-2 on mink farms between humans and mink and back to humans. *Science* 2021;371:172–7. 10.1126/science.abe5901 33172935 PMC7857398

[ref47] Mūrniece G, Šteingolde Ž, Cvetkova S et al. Prevalence of SARS-CoV-2 in domestic cats (*Felis catus*) during COVID-19 pandemic in Latvia. *Vet Med Sci* 2024;10:e1338. 10.1002/vms3.1338 38140758 PMC10951624

[ref48] O’Toole Á, Scher E, Underwood A et al. Assignment of epidemiological lineages in an emerging pandemic using the pangolin tool. *Virus Evol* 2021;7:1–9. 10.1093/ve/veab064 PMC8344591 34527285

[ref49] Oreshkova N, Moelnaar RJ, Vreman S et al. SARS-CoV-2 infection in farmed minks, the Netherlands, April and May 2020. *Euro Surveill* 2020;25:1–7. 10.2807/1560-7917.ES.2020.25.23.2001005 PMC7403642 32553059

[ref50] Pekar JE, Magee A, Parker E et al. The molecular epidemiology of multiple zoonotic origins of SARS-CoV-2. *Science* 2022;377:960–6. 10.1126/science.abp8337 35881005 PMC9348752

[ref51] Porter AF, Purcell DFJ, Howden BP et al. Evolutionary rate of SARS-CoV-2 increases during zoonotic infection of farmed mink. *Virus Evol* 2023;9:vead002. 10.1093/ve/vead002 36751428 PMC9896948

[ref52] Prince T, Smith SL, Radford AD et al. SARS-CoV-2 infections in animals: reservoirs for reverse zoonosis and models for study. *Viruses* 2021;13:1–14. 10.3390/v13030494 PMC8002747 33802857

[ref53] Qiu X, Liu Y, Sha A. SARS-CoV-2 and natural infection in animals. *J Med Virol* 2022;95:e28147. 10.1002/jmv.28147 36121159 PMC9538246

[ref54] Quinlan AR, Hall IM. BEDTools: a flexible suite of utilities for comparing genomic features. *Bioinformatics* 2010;26:841–2. 10.1093/bioinformatics/btq033 20110278 PMC2832824

[ref55] R&D, MGI Tech Bioinformatics . *MGI-tech-bioinformatics/SARS-CoV-2_Multi-PCR_v1.0. 13 Apr. 2020*. 2024. https://github.com/MGI-tech-bioinformatics/SARS-CoV-2_Multi-PCR_v1.0 (24 July 2025, date last accessed).

[ref56] Rabalski L, Kosinski M, Smura T et al. Severe acute respiratory syndrome coronavirus 2 in farmed mink (*Neovison vison*), Poland. *Emerg Infect Dis* 2021;27:2333–9. 10.3201/eid2709.210286 34423763 PMC8386773

[ref57] Rambaut A, Holmes EC, O’Toole Á et al. A dynamic nomenclature proposal for SARS-CoV-2 lineages to assist genomic epidemiology. *Nat Microbiol* published online 2020;5:1403–7. 10.1038/s41564-020-0770-5 32669681 PMC7610519

[ref58] Rasmussen TB, Qvesel AG, Pedersen AG et al. Emergence and spread of SARS-CoV-2 variants from farmed mink to humans and back during the epidemic in Denmark, June–November 2020. *PLoS Pathog* 2024;20:e1012039. 10.1371/journal.ppat.1012039 38950065 PMC11244769

[ref59] Ren W, Lan J, Ju X et al. Mutation Y453F in the spike protein of SARSCoV-2 enhances interaction with the mink ACE2 receptor for host adaptation. *PLoS Pathog* 2021;17:1–21. 10.1371/journal.ppat.1010053 PMC8601601 34748603

[ref60] Revell LJ . phytools 2.0: an updated R ecosystem for phylogenetic comparative methods (and other things). *PeerJ* 2024;12:e16505. 10.7717/peerj.16505 38192598 PMC10773453

[ref61] Richard M, Kok A, de Meulder D et al. SARS-CoV-2 is transmitted via contact and via the air between ferrets. *Nat Commun* 2020;11:1–6. 10.1038/s41467-020-17367-2 32641684 PMC7343828

[ref62] SAGO . *Independent Assessment of the Origins of SARS-CoV-2, Developed by the Scientific Advisory Group for the Origins of Novel Pathogens (SAGO)*. 2025. https://www.who.int/publications/m/item/independent-assessment-of-the-origins-of-sars-cov-2-from-the-scientific-advisory-group-for-the-origins-of-novel-pathogens (11 August 2025, date last accessed).

[ref63] Schindell BG, Allardice M, McBride JAM et al. SARS-CoV-2 and the missing link of intermediate hosts in viral emergence—what we can learn from other betacoronaviruses. *Front Virol* 2022;2:875213. 10.3389/fviro.2022.875213

[ref64] Sharun K, Dhama K, Pawde AM et al. SARS-CoV-2 in animals: potential for unknown reservoir hosts and public health implications. *Vet Q* 2021;41:181–201. 10.1080/01652176.2021.1921311 33892621 PMC8128218

[ref65] Smičius M, Olendraitė I, Bačelis J et al. Anthropozoonotic spillovers reveal sustained long-term cryptic circulation of SARS-CoV-2 within and between Lithuanian mink farms. *Virus Evol* 2026;12:veag014. 10.1093/ve/veag014 41994318 PMC13082393

[ref66] Starr TN, Greaney AJ, Hilton SK et al. Deep mutational scanning of SARS-CoV-2 receptor binding domain reveals constraints on folding and ACE2 binding. *Cell* 2020;182:1295–310.e20. 10.1016/j.cell.2020.08.012 32841599 PMC7418704

[ref67] Starr TN, Greaney AJ, Addetia A et al. Prospective mapping of viral mutations that escape antibodies used to treat COVID-19. *Science* 2021;371:850–4. 10.1126/science.abf9302 33495308 PMC7963219

[ref68] Tan CCS, Lam SD, Richard D et al. Transmission of SARS-CoV-2 from humans to animals and potential host adaptation. *Nat Commun* 2022;13:2988. 10.1038/s41467-022-30698-6 35624123 PMC9142586

[ref69] Temmam S, Vongphayloth K, Baquero E et al. Bat coronaviruses related to SARS-CoV-2 and infectious for human cells. *Nature* 2022;604:330–6. 10.1038/s41586-022-04532-4 35172323

[ref70] Tsueng G, Mullen JL, Alkuzweny M et al. Outbreak.info Research Library: a standardized, searchable platform to discover and explore COVID-19 resources. *Nat Methods* 2023;20:536–40. 10.1038/s41592-023-01770-w 36823331 PMC10393269

[ref71] Wan Y, Shang J, Graham R et al. Receptor recognition by the novel coronavirus from Wuhan: an analysis based on decade-long structural studies of SARS coronavirus. *J Virol* 2020;94:e00127-20. 10.1128/jvi.00127-20 31996437 PMC7081895

[ref72] Wang LG, Lam TTY, Xu S et al. Treeio: an R package for phylogenetic tree input and output with richly annotated and associated data. *Mol Biol Evol* 2020;37:599–603. 10.1093/molbev/msz240 31633786 PMC6993851

[ref73] Wei C, Shan KJ, Wang W et al. Evidence for a mouse origin of the SARS-CoV-2 omicron variant. *J Genet Genom* 2021;48:1111–21. 10.1016/j.jgg.2021.12.003 PMC8702434 34954396

[ref74] WHO . COVID-19 Weekly Epidemiological Update, Vol. 58. World Health Organization, 2022. 1–23. https://www.who.int/docs/default-source/coronaviruse/situation-reports/20210921_weekly_epi_update_58.pdf?sfvrsn=2ec52077_3&download=true

[ref75] Wickham H . Ggplot2: Elegant Graphics for Data Analysis. Springer Publishing Company, Incorporated, 2009. https://link.springer.com/book/10.1007/978-0-387-98141-3

[ref76] Wickham H, François R, Henry L et al. dplyr: A Grammar of Data Manipulation. 2025. https://dplyr.tidyverse.org/authors.html#citation (11 August 2025, date last accessed).

[ref77] Wilkinson SAJ, Richter A, Casey A et al. Recurrent SARS-CoV-2 mutations in immunodeficient patients. *Virus Evol* 2022;8:veac050. 10.1093/ve/veac050 35996593 PMC9384748

[ref78] Worobey M, Levy JI, Serrano LM et al. The Huanan Seafood Wholesale Market in Wuhan was the early epicenter of the COVID-19 pandemic. *Science* 2022;377:951–9. 10.1126/science.abp8715 35881010 PMC9348750

[ref79] Wu F, Zhao S, Yu B et al. A new coronavirus associated with human respiratory disease in China. *Nature* 2020;579:265–9. 10.1038/s41586-020-2008-3 32015508 PMC7094943

[ref80] Ye ZW, Yuan S, Yuen KS et al. Zoonotic origins of human coronaviruses. *Int J Biol Sci* 2020;16:1686–97. 10.7150/ijbs.45472 32226286 PMC7098031

[ref81] Yu G . Using ggtree to visualize data on tree-like structures. *Curr Protoc Bioinformatics* 2020;69:e96. 10.1002/cpbi.96 32162851

[ref82] Zhao J, Cui W, Tian BP. The potential intermediate hosts for SARS-CoV-2. *Front Microbiol* 2020;11:1–11. 10.3389/fmicb.2020.580137 33101254 PMC7554366

[ref83] Zhou P, Yang XL, Wang XG et al. A pneumonia outbreak associated with a new coronavirus of probable bat origin. *Nature* 2020;579:270–3. 10.1038/s41586-020-2012-7 32015507 PMC7095418

[ref84] Zhou J, Peacock TP, Brown JC et al. Mutations that adapt SARS-CoV-2 to mink or ferret do not increase fitness in the human airway. *Cell Rep* 2022;38:110344. 10.1016/j.celrep.2022.110344 35093235 PMC8768428

[ref85] Zrelovs N, Ustinova M, Silamikelis I et al. First report on the Latvian SARS-CoV-2 isolate genetic diversity. *Front Med* published online 2021;8:626000. 10.3389/fmed.2021.626000 PMC8055824 33889583

