# Supplementary material for: Evolution of SARS-CoV-2 caused infection in farmed minks: continuous surveillance of an 11-month outbreak at the largest Latvian mink farm
Source: Virus Evol. 2026 Jun 27;12(1):veag038. doi: 10.1093/ve/veag038 (PMC13367582; doi:10.1093/ve/veag038)
Supplement: Supplementary_materials_veag038 [file supplementary_materials_veag038.zip › Supplementary_Table_3.GISAID_supplemental_table_epi_set_260510vs.pdf]

Supplementary Appendix

All genome sequences and associated metadata supporting the findings of this study can be accessed through the persistent digital object identifier https://doi.org/10.55876/gis8.260510vs

In addition to the minted DOI, GISAID also communicates the aggregation of GISAID accession numbers (EPI_ISL_IDs) through the corresponding EPI_SET_260510vs identifier to facilitate both, the acknowledgment of all data contributors and the direct retrieval of the underlying data from GISAID used in this study.

hCoV-19 Virus Data Summary

GISAID Identifier	Digital Object Identifier	Number of individual viruses	Data Collection range	Number of countries/territories
EPI_SET_260510vs	https://doi.org/10.55876/gis8.260510vs	162	2021-04-07 to 2022-01-28	10
