# Supplementary material for: Evolution of SARS-CoV-2 caused infection in farmed minks: continuous surveillance of an 11-month outbreak at the largest Latvian mink farm
Source: Virus Evol. 2026 Jun 27;12(1):veag038. doi: 10.1093/ve/veag038 (PMC13367582; doi:10.1093/ve/veag038)
Supplement: Supplementary_materials_veag038 [file supplementary_materials_veag038.zip › Supplementary_Table_2.GISAID_supplemental_table_epi_set_250722ps.pdf]

SUPPLEMENTAL TABLE

Data Availability

GISAID Identifier:	EPI_SET_250722ps
DOI:	https://doi.org/10.55876/gis8.250722ps

All genome sequences and associated metadata in this dataset are published in GISAID’s EpiCoV database. To view the contributors of each individual sequence with details such as accession number, Virus name, Collection date, Originating Lab and Submitting Lab and the list of Authors, visit EPI_SET_250722ps

Data Snapshot

EPI_SET_250722ps is composed of 261 individual genome sequences.
The collection dates range from 2021-01-25 to 2022-01-28;
Data were collected in 1 countries and territories.
