# Supplementary figures and images for: Evolution of SARS-CoV-2 caused infection in farmed minks: continuous surveillance of an 11-month outbreak at the largest Latvian mink farm
Source: Virus Evol. 2026 Jun 27;12(1):veag038. doi: 10.1093/ve/veag038 (PMC13367582; doi:10.1093/ve/veag038)

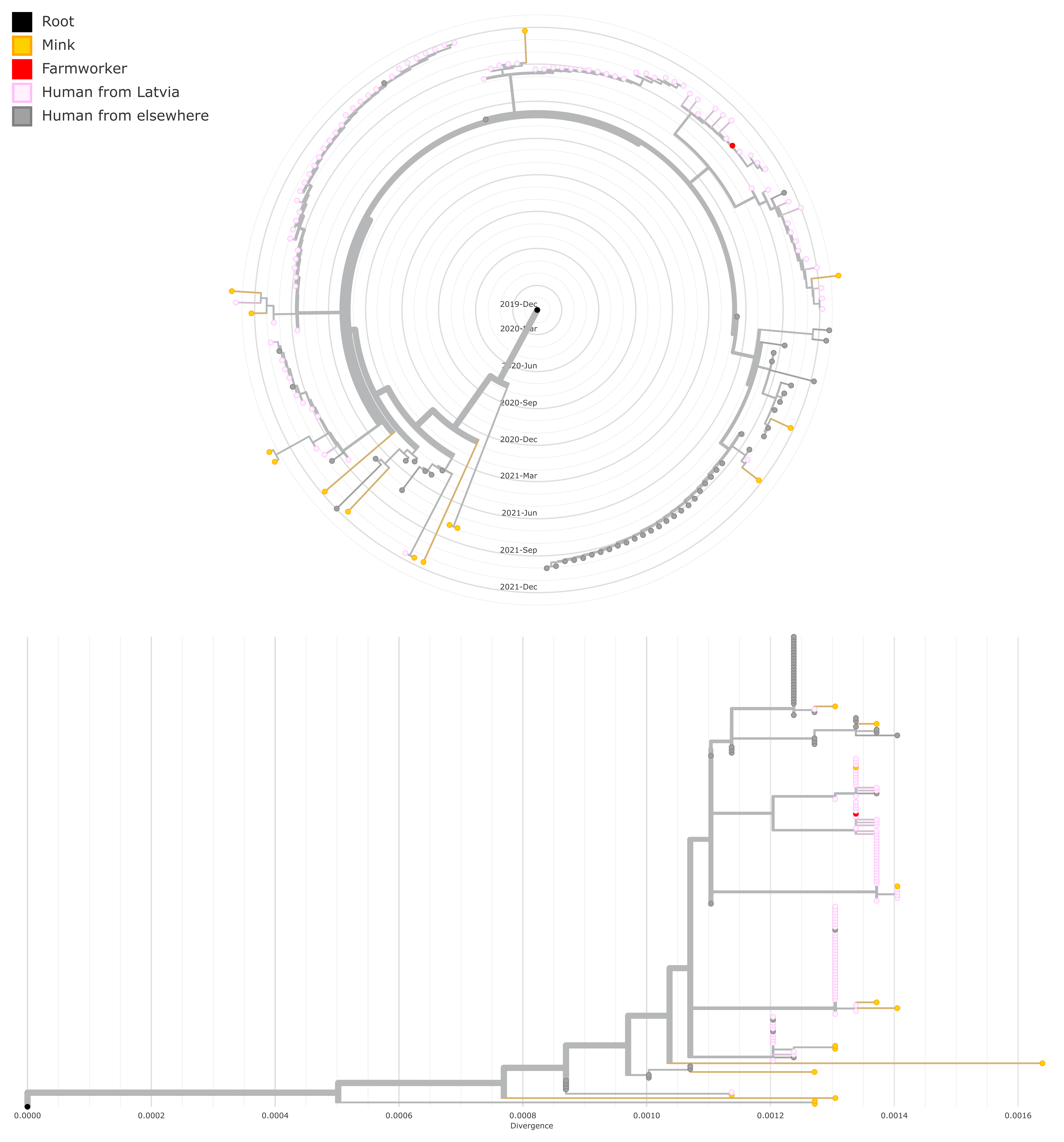

Supplement: Supplementary_materials_veag038 [file supplementary_materials_veag038.zip › Supplementary_Figure_4.GK_clade_sequence_context.png]

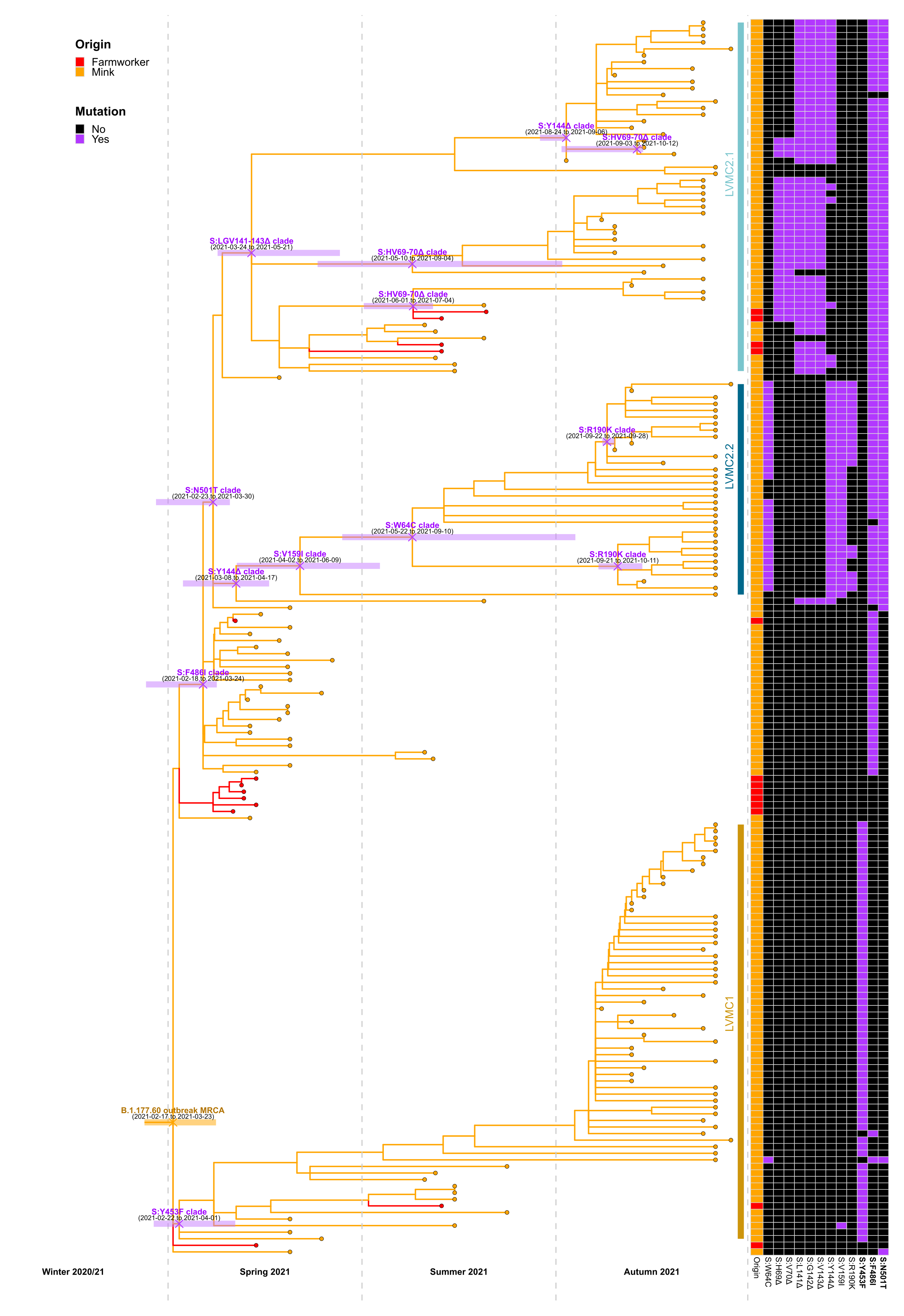

Supplement: Supplementary_materials_veag038 [file supplementary_materials_veag038.zip › Supplementary_Figure_3.Timetree_with_MRCA_annotations_of_interest.png]

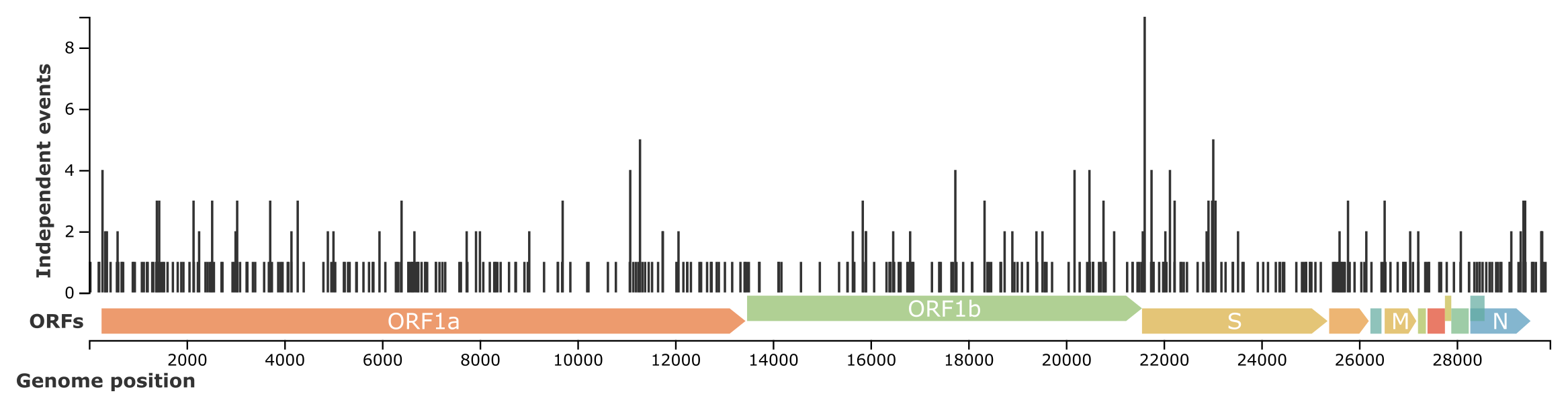

Supplement: Supplementary_materials_veag038 [file supplementary_materials_veag038.zip › Supplementary_Figure_2.Mutational_landscape.png]

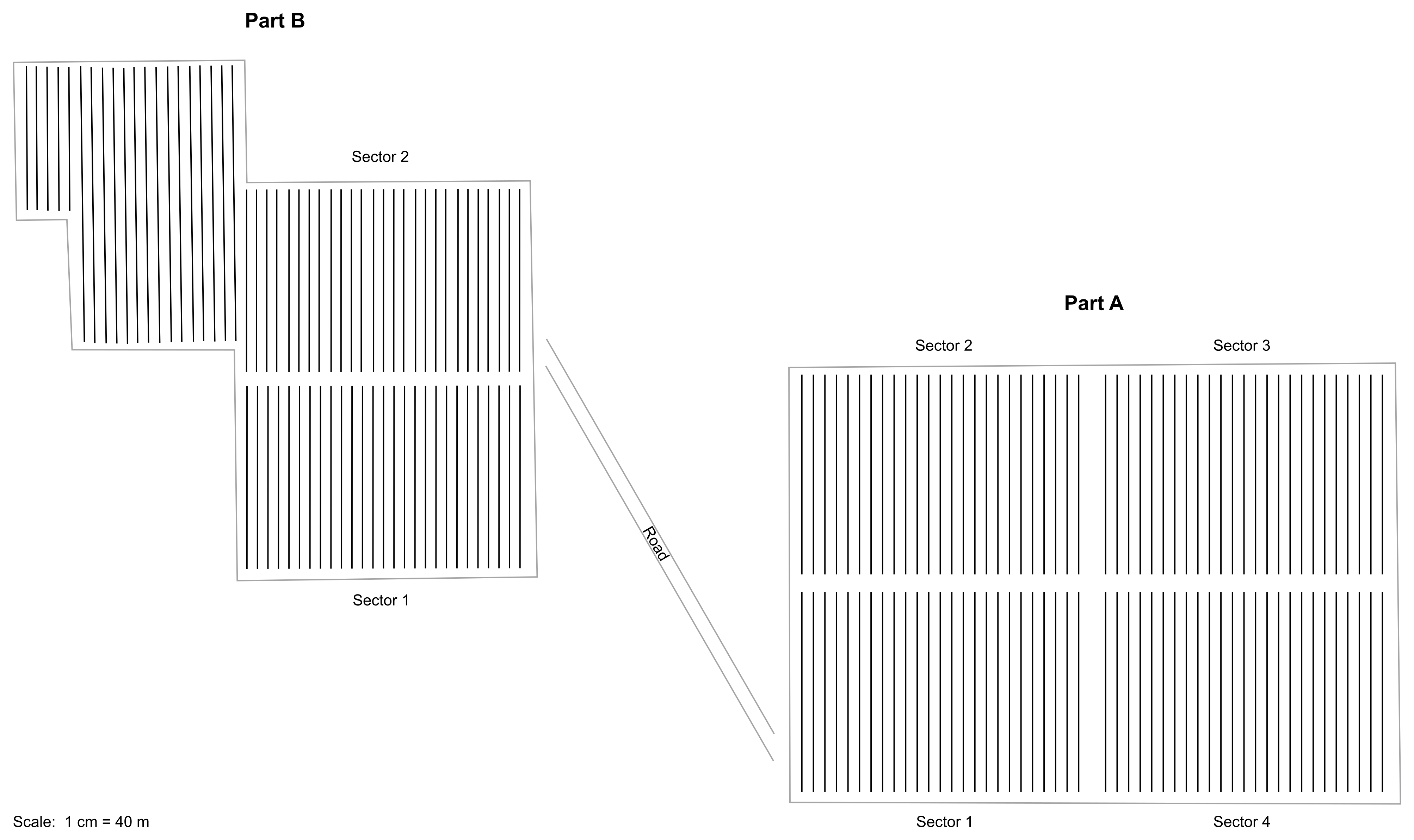

Supplement: Supplementary_materials_veag038 [file supplementary_materials_veag038.zip › Supplementary_Figure_1.Schematic_mink_farm_layout.png]
